# Cation-Surface Interactions During Electrocatalytic Hydrogen Evolution Probed by Surface X‑ray Diffraction

**DOI:** 10.1021/acsphyschemau.5c00152

**Published:** 2026-03-03

**Authors:** Mariana C. O. Monteiro, Leon Jacobse, Arthur M. V. Hagopian, Vedran Vonk, Simon Chung, Sheena Louisia, Alexander Meinhardt, Elif Öykü Alagöz, Xin Deng, Beatriz Roldan Cuenya, Katharina Doblhoff-Dier, Marc T. M. Koper, Andreas Stierle

**Affiliations:** † Department of Interface Science, Fritz-Haber Institute of the Max Planck Society, Faradayweg 4-6, 14195 Berlin, Germany; ‡ Leiden Institute of Chemistry, 4496 Leiden University, P.O. Box 9502, 2300 RA Leiden, The Netherlands; § Centre for X-ray and Nano Science CXNS, 28332 Deutsches Elektronen-Synchrotron DESY, Notkestrasse 85, D-22607 Hamburg, Germany; ∥ Electrical and Computer Engineering, 3990 Rice University, 6100 Main Street, Houston, Texas 77005, United States; ⊥ Fachbereich Physik, Universität Hamburg, Jungiusstraße 11, 20355 Hamburg, Germany

**Keywords:** double-layer structure, alkali cations, specular crystal truncation rod, hydration shell structure, hydrogen evolution reaction

## Abstract

The electrocatalytic production of hydrogen is pivotal for the sustainable generation of hydrogen fuel and hydrogen as reducing agent. Metal cations in alkaline electrolytes can facilitate or significantly impede this reaction, depending on electrolyte pH, catalyst surface, cation identity, and concentration. Still, the underlying mechanisms governing these effects remain elusive, in part due to the lack of direct characterization of how cations interact with the electrocatalyst surface and interfacial water. Here, using Surface X-ray Diffraction combined with ab initio Molecular Dynamics, we elucidate the formation of a cation layer at the electrochemical interface during hydrogen evolution on a hexagonally reconstructed Au(100) model catalyst under varying pH, potential, cation concentration, and electrolyte composition. Specular Crystal Truncation Rod measurements show that as the potential becomes more negative, Cs^+^ cations increase in surface coverage and move progressively closer to the interface, revealing that the positions of the inner- and outer-Helmholtz planes continuously shift with potential. Slightly smaller cation-to-surface distances and higher coverages are observed in alkaline media compared to acidic environments. Ab initio Molecular Dynamics simulations reveal that the Cs^+^ ions move into the first water layer as they approach the surface at more negative potentials. No water molecules can then reside between the ions and the Au surface, leading to a broken solvation symmetry and an opening angle. This opening is initially established by an orientation and slight distortion of the solvation shell, while at shorter ion-surface distances the ion sheds ∼10% of its solvation, enabling a closer approach to the surface. Finally, in Li^+^/Cs^+^ electrolyte mixtures at pH 3, we observe the preferential accumulation of Cs^+^ at the electrochemical interface, albeit in a more disordered fashion compared to the Li^+^-free situation. This is expected to enhance interfacial mobility and influence electrocatalytic activity beyond traditional “structure making/breaking” descriptions.

## Graphical abstract

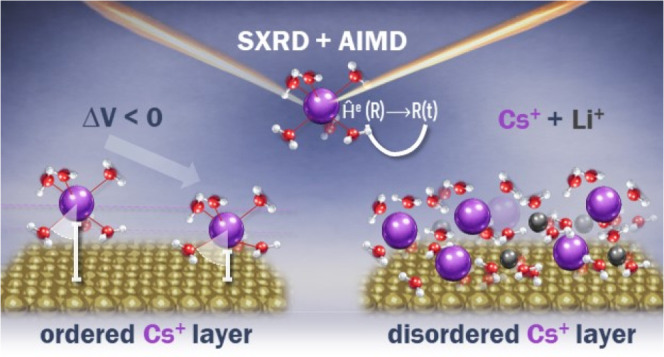

## Introduction

Electrocatalysis is crucial for advancing sustainable energy production toward a fossil fuel-free electrified society. The hydrogen evolution reaction (HER), a fundamental process within the field, is notably influenced by cationic species in the electrolyte in neutral and alkaline media, although their exact role is still debated.^1^ This has prompted increased attention toward optimizing the electrolyte composition to enhance HER performance.^2–12^ Despite significant experimental and theoretical efforts, direct evidence of the structure and behavior of the electrochemical double-layer (EDL) during HER and other reactions remains scarce due to the limited number of techniques sensitive to the double-layer structure. Furthermore, recent studies indicate deviations from classical EDL theories under certain conditions, increasing the complexity of understanding electrocatalytic systems without operando characterization.^13–15^ Resolving the electrochemical interface structure under HER conditions is thus essential for comprehending the interaction of electrolyte species with electrode surfaces and designing improved catalytic systems.

One of the earliest reports on the effects of cations on HER in acidic media by Herasymenko and Šlendyk suggested a simple competitive Langmuir adsorption model to explain activity trends.^16^ Later works^17–22^ have also suggested that cations adsorb on the electrode surface under HER conditions, but with limited direct experimental evidence. In contrast, Huang et al. suggests that cations affect the HER kinetics by altering the interfacial water structure and H-bonding network to different extents depending on their hydration energy.^23^ Adding to the complexity, cation and pH effects on HER cannot be treated separately, especially in alkaline media. On gold electrodes, increasing the (local) electrolyte pH from 11 to 13 is proposed to result in higher cation concentrations near the surface, facilitating the water reduction reaction.^24^ However, at bulk pH ≥ 13, cations start to strongly inhibit HER. The precise mechanism behind this inhibition remains unresolved. Still, the negative reaction order on cation concentration follows the sequence K^+^ < Na^+^ < Li^+^,^25^ suggesting that the ion’s hydration energy could play a role. In later work, we observed that this inhibition is not only dependent on the pH and cation identity, but also on the nature and orientation of the electrode surface: the HER inhibition regime on platinum is observed at lower alkalinity and cation concentrations than on gold, and is also highly sensitive to the platinum surface facet.^11,12,25^ Interestingly, alkaline water electrolyzers typically utilize highly concentrated KOH as electrolyte due to its optimum conductivity and solubility. However, the works discussed here clearly demonstrate that weakly hydrated K^+^ species contribute to a decline in catalytic activity on both Pt and Au electrodes in strong alkaline solutions. Indeed, for one of the best catalysts known for alkaline HER, that is PtRu, LiOH is the optimal electrolyte.^12^

Direct experimental evidence of the pH and ion identity dependence of cation–surface interactions is desired to help interpreting catalytic trends for the HER and beyond.^8,11,24,25^ Traditional techniques such as cyclic voltammetry, and different spectroscopies have been extensively employed in this regard. However, these methods provide limited information about e.g., the ordering of ions at the EDL. Surface X-ray Diffraction (SXRD) has been shown to be a powerful technique for probing the local atomic scale structure of electrochemical interfaces.^26,27^ In SXRD, in addition to the Bragg peaks originating from the bulk crystal lattice, the presence of a sharp (ordered) surface leads to finite diffracted intensity along so-called Crystal Truncation Rods (CTRs), which extend in reciprocal space along the surface normal direction. Specifically, the specular CTR (0, 0, L), extends only along the surface normal component of the momentum transfer, thereby probing the electron density ordering only along this direction. As such, also layers without in-plane ordering contribute to the specular rod.^28–30^ Specular CTR measurements aiming to resolve the EDL structure have so far been performed mainly on gold^30,31^ and platinum^28,29,32–35^ electrodes at potentials close to the electrode potential of zero charge (PZC) or higher, or/and at constant pH.^8,11,24^

In this work, we used SXRD to investigate how pH, potential, and cation identity influence cation–surface interactions, focusing on the Cs^+^ interaction with Au(100) as a model system. These experiments are enabled by the fact that Au(100) exhibits a wide potential and pH window where its (hexagonally reconstructed) surface is stable while also no specific adsorption of electrolyte species takes place.^36,37^ Furthermore, fundamentally similar alkaline hydrogen evolution trends are observed for gold and more active surfaces as Pt—with the main differences coming from the potential, pH and cation concentration at which they manifest. We studied this system at pH 3 and 13, and examined the effect of adding Cs^+^ to Li^+^-containing electrolytes. A Cs^+^ layer forms under all applied potentials and pH conditions, with slightly higher coverage in alkaline media but similar cation-to-surface distances. Mixing Cs^+^ with Li^+^ results that Cs^+^ preferentially occupies the EDL however in a more disordered fashion, indicating significant changes in EDL structure and highlighting the role of nonelectrostatic interactions. Ab Initio Molecular Dynamics (AIMD) simulations provide insight into Cs^+^ solvation at the distances observed by SXRD, showing that at the most negative potentials, Cs^+^ ions approach the surface closely enough to shed part of their solvation shell, leaving no water molecules between the ion and electrode. Interestingly, while the number of water molecules near the surface remains largely unaffected by ion proximity, the population slightly further away decreases. Together, experiments and simulations reveal a dynamic EDL structure beyond the classic inner- and outer-Helmholtz plane model, with implications for HER and other electrocatalytic reactions.

## Methods

### Experimental Section

In situ surface X-ray diffraction experiments were conducted at the in situ and nano X-ray diffraction beamline of PETRA III, *P*23, at the Deutsches Elektronen Synchrotron (DESY), employing a photon energy of 20 keV. A Lambda 750k GaAs detector was used with a pixel size of 55 × 55 μm^2^. The following gold lattice parameters were used: *a* = *b* = *c* = 4.0782 Å; α = β = γ = 90°. The specular CTR profiles were determined by performing detector rocking curves at various θ/2θ positions to enable subtraction of a suitable background. The data processing^38^ (intensity correction, signal integration, etc.), which renders structure factors, was performed using home-written scripts in Wavemetrics Igor Pro and the ANAROD package was used to fit the structural models to the data.^39^ Further experimental details (electrochemical cell, sample preparation, chemicals) can be found in the Supporting Methods in the Supporting Information (SI).

### Computational

All AIMD simulations were performed with CP2K version 2022.1. The simulation cell consists of a hexagonally reconstructed Au(100) slab (with the reconstruction approximated in a 5 × 6 surface unit cell as suggested in ref. 59), 154 water molecules and a vacuum interface.^40^ The water structure is pre-equilibrated for the half cell (5 × 3 surface) at the force-field level. Then ions are inserted into the unit (half) cell and an AIMD equilibration of 15 ps is performed. The system is then doubled (to give the above dimensions), and further equilibrated for 5 ps. Data is collected for another 15 ps. All AIMD simulations were performed using the PBE functional with D3 corrections. We used a plane wave cutoff energy of 400 Ry, a DZVP-MOLOPT-SR-GTH basis set and represent the atomic cores by GTH-PBE-qi pseudopotentials, where *i* = 6, 1, 11, 9 electrons for O, H, Au, and Cs, respectively. Further details can be found in the Supporting Methods in the SI.

## Results

### Effect of Potential and pH

The Au(100) sample used in this work was prepared by flame annealing in order to thermally reconstruct the surface, forming a 20 × 5 hexagonal top layer (*hex*-Au­(100), see Figure S1).^27,41^ The atom density in this reconstructed layer is 20% higher compared to the bulk-terminated surface, which leads to an out-of-plane expansion and enhanced corrugation.^37^ The cyclic voltammogram (CV, see Figure S2) recorded in the SXRD cell in 0.1 M CsOH shows the characteristic peak associated with the lifting of the hexagonal reconstruction layer between 0 and 0.2 V versus Ag/AgCl, above which potential the 1 × 1 surface structure is formed.^41^ In order to reliably compare cation–surface interactions at different conditions, we always perform experiments on the same, freshly prepared surface termination (*hex*-Au­(100)), and make sure that this surface is stable throughout the measurement duration. For each measurement, the surface structure and stability are evaluated by measuring the in-plane diffraction signal (Figure 1a) corresponding to the gold hexagonal reconstruction surface rod before, as well as after applying a potential sequence. An example of such measurements can be seen in Figure 1b, showing azimuthal in-plane scans of the *hex*-Au­(100) electrode recorded at *L* = 0.045 at a constant potential (−0.5 V vs Ag/AgCl in 0.1 M CsOH) before and after cyclic voltammetry. The comparable signal intensity before and after certifies that the hexagonal layer is stable enough during the experiment. The observed shift of the maximum peak intensity is due to the formation of slightly rotated domains (by ∼0.3°), in agreement with previous reports.^37^ Note that, if the reconstruction would have been lifted during the experiment, these rotated domains would dominate the diffraction pattern.^37^

**Figure 1:**
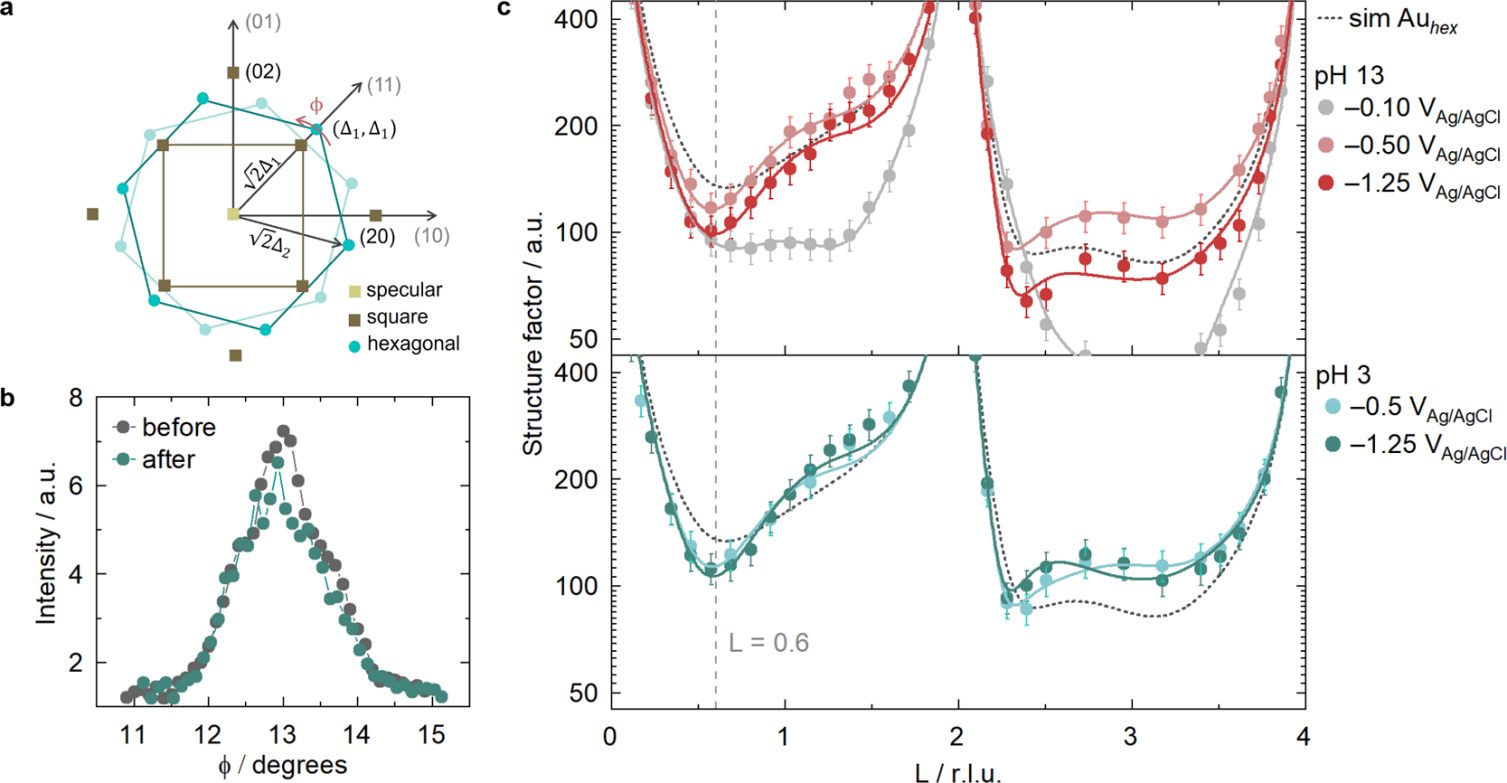
Specular CTRs at different pH. (**a**) Schematics of the in-plane diffraction pattern of the reconstructed (hexagonal) and unreconstructed (square) Au(100) surface. Each point represents a rod of scattering normal to the surface. The characteristic wave vectors along the [110] and the rotated directions are indicated. (**b**) Azimuthal (Φ) in-plane scan centered around [Δ_1_, Δ_1_, 0] recorded at *L* = 0.045, in 0.1 M CsOH (pH 13) at −0.5 V versus Ag/AgCl before and after specular CTR measurements; (**c**) specular CTRs of Au(100) recorded in 0.1 M CsOH (pH 13) and 0.08 M CsClO_4_ + 1 mM HClO_4_ (pH = 3) at different constant applied potentials, together with fits of the experimental data to the structural model from Figure 2c. The black dotted line is the simulated specular CTR of a “bare” *hex*-Au­(100) surface (without a Cs^+^ layer), obtained using the parameters from Supporting Table 1 in the Supporting Discussion and the structural model shown in Figure 2a. The potentials correspond to the following potentials on the reversible hydrogen electrode scale: pH 13 (0.864, 0.464, −0.286 V vs RHE) and pH 3 (−0.126, −0.876 V vs RHE).

To enable a quantitative analysis of the interfacial cation layer during HER, we performed specular CTR measurements at different constant potentials, both at pH 3 and 13 in electrolytes containing Cs^+^ cations. Cs^+^ was chosen as it has the highest electron density of the alkali metals, leading to enhanced scattering of the X-rays and consequently better contrast in the recorded signal. Figure 1c shows the specular CTR data obtained, together with the simulated specular CTR of a “bare” *hex*-Au­(100) surface (without a Cs^+^ layer), obtained using the parameters from Supporting Table 1 and the structural model shown in Figure 2a. The presence of a Cs^+^ layer with a well-defined out-of-plane position leads to clear changes in the signal intensity (Figure 1c). As an additional control experiment, we have also recorded the specular CTR at −0.1 V versus Ag/AgCl at pH 13 (shown in gray). At this potential, the hexagonal reconstruction is already partially lifted, evidenced by a sharp decrease in signal intensity in Figure 1c due to the adatom island formation on the unreconstructed surface,^41^ and the nearly total loss of the surface rod signal (Figure S3 in the Supporting Information (SI)). For a quantitative analysis, a structural model of a hexagonally terminated Au(100) surface with one Cs^+^ layer was fitted to the data. For easier visualization the 20 × 5 unit cell (Figure S1 in the SI) is approximated in Figure 2a with a 1 × 5 structure. This profile view of the *hex-*Au­(100) emphasizes the buckling of the Au surface atoms due to the lattice mismatch between the surface and bulk atomic structure. Importantly, the specular CTR signal is not sensitive to the in-plane structure of the surface reconstruction, but merely to its out-of-plane electron density and (average) roughness. This means that for the analysis, we can reduce the complex (20 × 5) unit cell, to a simple (1 × 1) structure.^37^ For the Cs^+^ and Au_*hex*_ atomic layers the displacement, occupancy, and root-mean-square displacements (Debye–Waller factors, DW) were fitted. It should be noted that the DW factor of the Au_*hex*_ layer is dominated by the enhanced corrugation due to the increased atom density within this layer, and is therefore significantly larger than that of the underlying layers. By systematically simulating the effect of different structural parameters on the specular CTR (see Figure S5 in the SI), it is concluded that changes in the interlayer distances can easily be distinguished from changes in the occupancy or the DW factors as these affect the CTR in a different manner: changes in the Au–Cs^+^ distance would mainly lead to (additional) asymmetry around the Bragg peaks, whereas an increase in Cs^+^ coverage would cause the intensity between the Bragg peaks to fall off sharply at ∼*L* < 1 (see Figure S5 in the SI). Since occupancies and the DW parameters are largely correlated, we fitted the Cs^+^ DW factors for the data at pH 13 (which resulted in values ∼15 for the data recorded both at −0.5 and −1.25 V vs Ag/AgCl (Figure S6a)) and used this value when fitting the data at pH 3. For the Au_2_ atoms, only the DW factor was fitted, fixing the occupancy at unit. For the Au_sur1_ and Au_bulk_ atoms (see Figure 2a), the occupancy was fixed at unity and the thermal Debye–Waller factors at the bulk room temperature value of 0.64 ^37^ and 0.63 ^42^ Å^2^, respectively.

**Figure 2:**
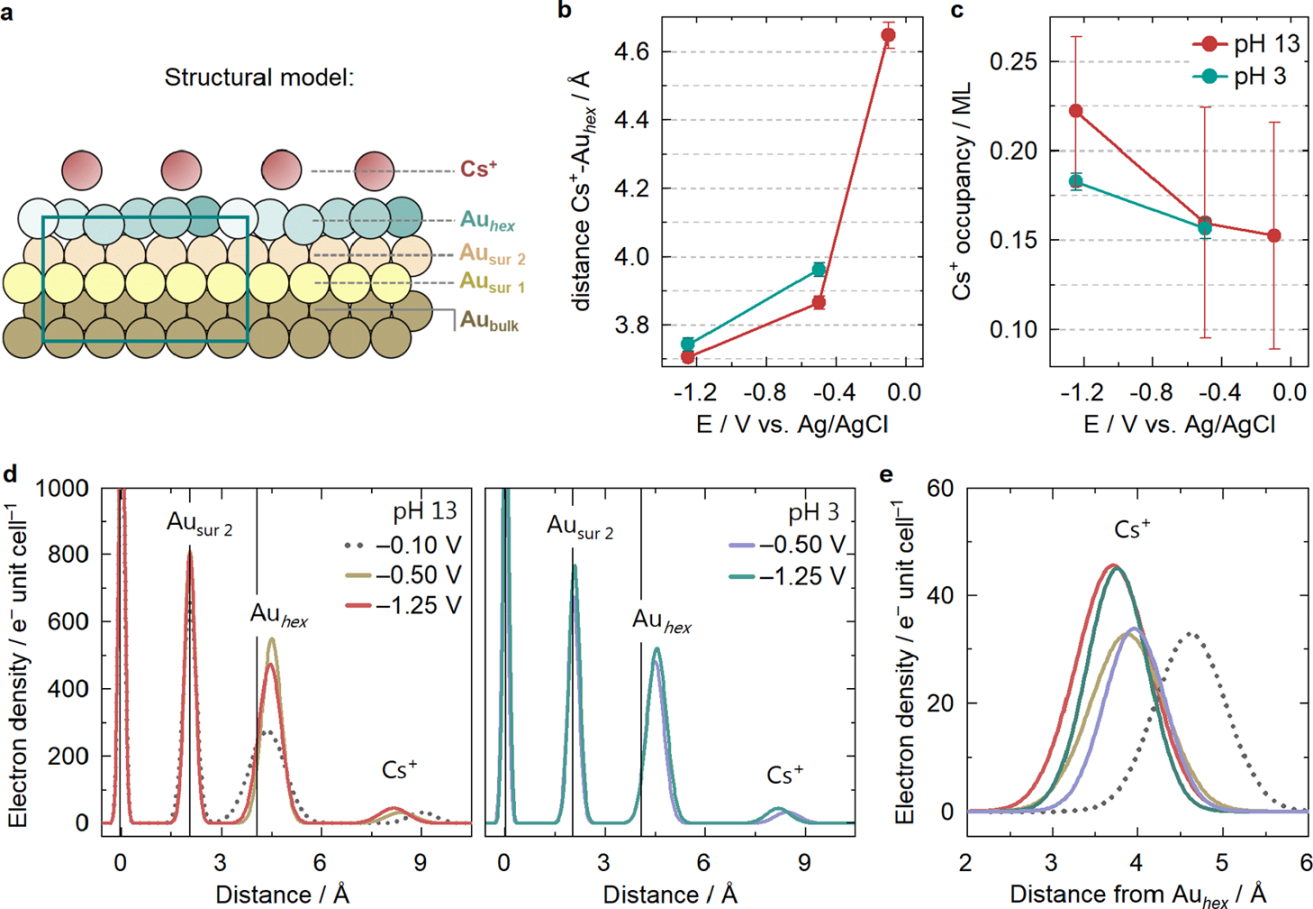
Fit results. (**a**) Structural model of the 20 × 5 hex-Au(100) surface, approximated by a 5 × 1 surface unit cell for easier visualization, depicting bulk, surface (sur1, sur2), and reconstruction (Au_*hex*_) layers as well as a layer of Cs^+^; (**b**) Cs^+^-surface distance and (**c**) Cs^+^ occupancy at different pH and potentials, obtained from fitting the data from Figure 1 to the structural model depicted in (a). (**d**) Z-projected electron density obtained from fitting the (0 0 L) CTRs at different pH together with (**e**) the out-of-plane Cs^+^ electron density; all fit results are available in Supporting Table 2, in the Supporting Discussion.

The fits are plotted together with the data in Figure 1c. The CTRs at both acidic and alkaline pH can be described very well with our relatively simple model, confirmed by the close to unit normalized chi square values obtained (χ^2^, Table S2 in the SI). Satisfactory fits could not be obtained when omitting the Cs^+^ layer from the model—not even when including water molecules in the model instead. All fit results are shown in Supporting Table 2 with the respective standard errors. The most relevant fitting parameters as a function of pH are summarized in Figure 2b,c, namely the Cs^+^ occupancy (in ML) and the Cs^+^-Au_hex_ distance. The Cs^+^-Au_hex_ distance decreases with more negative potential and reaches 3.71 ± 0.01 Å at −1.25 V versus Ag/AgCl. This may be explained by the increasingly negative surface charge exerting a stronger attractive force on the ions. At pH 13 the Cs^+^-Au_hex_ distance found at a given potential versus Ag/AgCl is slightly shorter than that found at pH 3. The reason for this small difference is not fully clear, but could lie in the fact that the double layer capacitance (and hence the surface charge) can depend on the electrolyte composition.

Consistent with the increasingly negative surface charge at potentials negative of the potential of zero charge (PZC), the Cs^+^ coverage increases when going from −0.5 V versus Ag/AgCl to −1.25 V versus Ag/AgCl. This increase is stronger at pH 13 than at pH 3. This difference in behavior between pH 13 and pH 3 is significant in spite of the relatively large error bars in the data for pH 13, for two reasons: (i) the Cs^+^ DW factors (which cause most of the uncertainty) remain constant over the potential range studied for pH 13; (ii) at −1.25 V, at which the Cs^+^-Au_hex_ distance is very similar at both pH, the difference in signal intensity around *L* = 0.6 must stem from a higher Cs^+^ coverage. Changes in the interfacial electric field strength could explain the higher Cs^+^ coverage at pH 13 at −1.25 V versus Ag/AgCl, as the surface electric field is expected to depend on the difference between the applied potential and the potential of zero charge PZC, and the capacitance. While, at any given potential on the Ag/AgCl scale, the difference between the applied potential and PZC is expected to be pH-independent (as the PZC is also expected to constant on an absolute, pH-independent scale such as the Ag/AgCl scale), the capacitance may still be pH dependent, affecting the interfacial electric field and hence the expected surface-near ion concentration. A higher Cs^+^-coverage in alkaline media could also arise from the fact that, at pH 3, hydronium (H_3_O^+^) can assist in the screening of the negative charge, which is not the case at pH 13. Such an effect would, however, be expected over the entire potential range, which is inconsistent with our measurements. What can explain our experimental observations, though, is the fact that, at pH 13 and −1.25 V versus Ag/AgCl water reduction will occur, leading to the production of OH^–^ at the interface. If these cannot be transported away from the interface fast enough, Cs^+^ will be attracted toward the surface to maintain charge neutrality. As such, this effect should be stronger at higher potential/higher current. At pH 3, at −0.5 V HER will primarily occur via proton reduction while at −1.25 V vs Ag/AgCl via water reduction. The higher proton concentration in the bulk accelerates the consumption of OH^–^ leading to a smaller pH gradient,^43^ and consequently a lower Cs^+^ coverage at larger overpotential.

Figure 2d shows the *z*-projected electron density obtained from analyzing the specular CTR, and Figure 2e a comparison of only the Cs^+^ electron density at different pH and potentials. At both pH values, the peaks from the Au_2_ and Au_hex_ vary only slightly with potential as long as the reconstruction is not lifted. These variations can be attributed to a slight expansion of the Au_hex_ layer (Figure S6c) and the expected small changes in DW and/or occupancy. The relative occupancies obtained between the Au_hex_ and Au_2_ layer are in perfect agreement with those expected for a hexagonal reconstruction (i.e., ∼21% additional surface atoms expected in comparison to a bulk terminated (100) surface (Figure S6b)).^37^ At −0.1 V versus Ag/AgCl at pH 13 (0.864 V vs RHE), the partial lifting of the hexagonal reconstruction is captured, with a sharp increase of the Au_hex_ DW factor to 22 ± 3 due to the rougher 1 × 1 structure formed, a decrease in the Au_hex_ occupancy to 1.12 ML, and a decrease in the Au_*hex*_-Au_2_ displacement (Figure S6c).

Changes in the Cs^+^ layer at the *hex-*Au­(100) were investigated during potential cycling. Since the measurement of the full specular features is not possible at reasonable scan rates, we measured the specular CTR integrated intensity at *L* = 0.6 during CVs between −0.5 and −1.25 V versus Ag/AgCl (Figure 3) at pH 3 and 13, at two different scan rates (2 and 10 mV/s). Based on simulations (see Figure S5), the highest sensitivity to the presence of cations is expected at this reflection. The signal displayed in Figure 3 is normalized to the intensity measured at −0.5 V. As expected from the full CTR measurements (Figure 1c), significant changes in the reflected intensity are observed with potential cycling. The decrease in signal intensity with decreasing potential matches well with the results discussed above, as, according to our simulations, a decrease in signal intensity correlates with an increasing coverage and/or a decreasing Cs^+^-to-surface distance. In the absence of metallic cations in the electrolyte, such a behavior is not observed for either the unreconstructed and the *hex*-Au­(100) surface, as studied by Ocko et al.^37^ The intensity changes are fully reversible as a function of potential both at pH 13 and pH 3, also seen by the good fit of the data using a simple triangular waveform (black lines). This reversibility is present independently of whether the gold is cycled at 2 mV s^–1^ or 10 mV s^–1^. Importantly, if a (partial) lifting of the reconstruction would occur, the potential dependency would be exactly opposite and strongly irreversible^37^ (see also Figure 1c). This measured signal must thus originate from the formation of an ordered Cs^+^ layer moving to and from the electrode surface rather than from the gold substrate itself.

**Figure 3:**
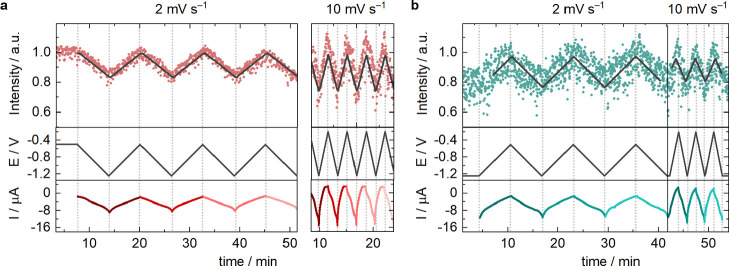
Time scans recorded at (0, 0, 0.6) during cyclic voltammetry (**a**) in CsOH (pH 13) at 2 and 10 mV s^–1^, (**b**) in CsClO_4_ (pH 3) at 2 and 10 mV s^–1^. The solid lines represent triangular waveforms fitted to the data. The middle panels show the applied potentials and the bottom panels the electrochemical current measured during the same experiment. The potential is reported versus Ag/AgCl.

Considering the radius of solvated (∼3.5 Å) and desolvated (∼1.8 Å) Cs^+^ ions,^44,45^ the experimental ion-surface distances (∼3.7 Å at −1.25 V vs Ag/AgCl; Figure 2b), suggest that the Cs^+^ ions must be partially desolvated at the electrified interface, in agreement with previous spectroscopic and theoretical evidence.^23,44,46–49^

### Double-Layer (dis)­ordering in Li^+^ + Cs^+^ Electrolyte Mixtures

Adding small amounts of Cs^+^ to a Li^+^-based electrolyte has been previously observed to enhance the HER activity of polycrystalline gold.^44^ To further understand the Cs^+^ layer formation and this effect, we used SXRD to study if, in such a mixed electrolyte, Cs^+^ preferentially occupies the EDL. For these experiments, 0.1 M LiClO_4_ adjusted to pH 3 was used as a background electrolyte, such that enough Li^+^ cations are present to screen the negatively charged surface. We have then added different concentrations of Cs^+^ to the electrolyte (5 and 10 mM) and recorded the specular CTR intensity at *L* = 0.6 during cyclic voltammetry (similar to Figure 3). Lithium atoms scatter X-rays more than two orders of magnitude weaker than Cs^+^, which is below the signal-to-noise ratio of these experiments. Indeed, in the presence of only Li^+^ no significant change in the integrated intensity is observed (Figure 4a), similar to when there is no cation in the electrolyte^37^ This would also be the case upon addition of e.g. a few mM Li^+^. On the other hand, when 5 mM CsClO_4_ is added to the electrolyte (pH = 3, kept constant), a decrease in signal intensity is observed as the electrode is cycled to −1.25 V versus Ag/AgCl, indicating the presence of Cs^+^ in the EDL. This remains reversible during the 5 cycles recorded, similarly to what was observed in Figure 3 for the pure CsClO_4_ electrolyte. The intensity decrease we observed in the pure 0.1 M Cs^+^-containing electrolyte at pH 3 is of approximately 20% (Figure 3b). Consequently, if the interfacial cation distribution were to follow the bulk ion ratio (i.e., Li^+^/Cs^+^ = 100:5), an intensity decrease of only 1% would be expected for the 0.1 M Li-electrolyte with 5 mM of Cs^+^. However, Figure 4a shows a much higher decrease in the integrated intensity, namely ∼10%, suggesting that despite having enough Li^+^ cations in the electrolyte to screen the negative surface charge as well as having a large excess of Li^+^ compared to Cs^+^, Cs^+^ preferentially goes to the interface. The latter is further supported by the fact that a doubling of the Cs^+^ concentration does not lead to further decrease in the CTR intensity.

**Figure 4:**
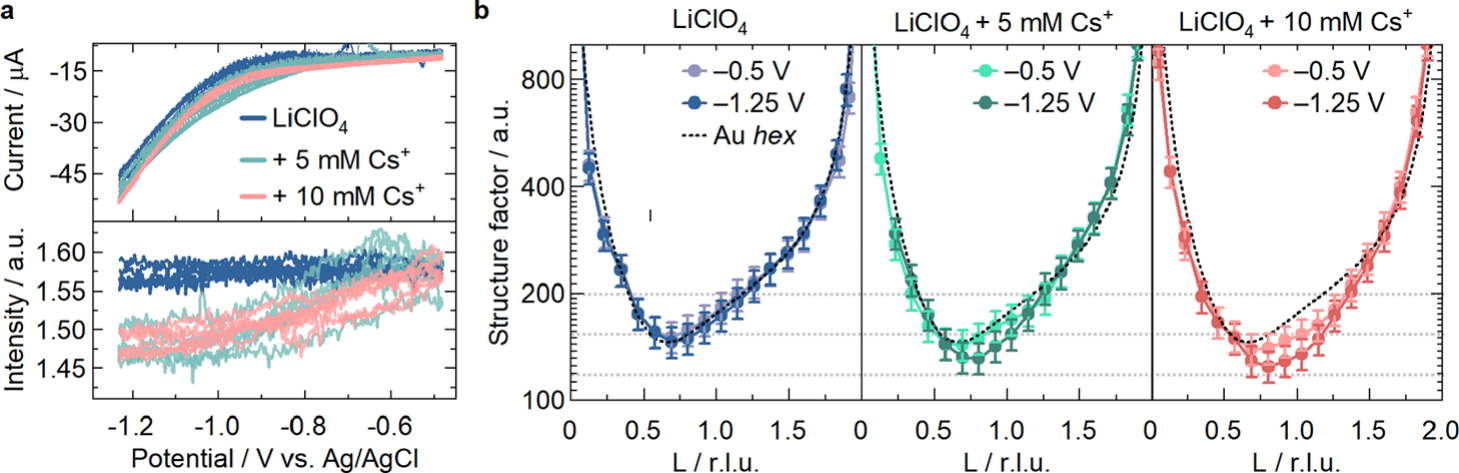
Specular measurements in electrolyte mixtures. (**a**) Integrated intensities recorded at (0, 0, 0.6) during five CVs in pure LiClO_4_ and with the addition of 5 and 10 mM of CsClO_4_. The electrolyte pH was kept constant (pH = 3) by the addition of 1 mM HClO_4_. (**b**) Specular CTR in the same electrolytes as a function of potential. The black dotted line is the simulated specular CTR of a “bare” *hex*-Au­(100) surface (without a Cs^+^ layer), obtained using the parameters from Supporting Table 1 in the Supporting Discussion. The potential is reported versus Ag/AgCl.

To quantitatively characterize the properties of the Cs^+^ layer in the presence of the Li^+^-background mixed electrolytes, we have performed specular CTR measurements in the range of the first minimum (0 < L < 2), where the signal is most sensitive to changes happening specifically in the cation layer. Figure 4b shows the specular CTRs recorded at two different potentials in pure 0.1 M LiClO_4_, and with the addition of 5 and 10 mM CsClO_4_, all at bulk pH = 3. As expected from the low scattering intensity of Li^+^, these data overlap perfectly with the simulated intensity for the Au_hex_-terminated surface. In contrast, in the presence of Cs^+^, the signal systematically decreases as the potential is more negative. Based on Figures 1c and 2 this clearly signifies changes in the atomic structure of the Cs^+^ layer. However, an attempt to fit the CTRs shown in Figure 4b with the model from Figure 2a (including a Cs^+^ layer) did not lead to satisfactory fits. This means that despite Cs^+^ being present at the interface (seen by the decrease in signal intensity in Figure 5a), in the presence of Li^+^ it does not form a layer with enough out-of-plane order to be fitted. This is in clear contrast to the observations made in the pure CsClO_4_ or CsOH electrolytes (Figure 1c). A direct comparison can be seen in Figure S8a, which clearly shows the differences in the CTR signal intensity between the pure and mixed electrolytes. Furthermore, subtle changes in the substrate cannot be completely excluded. However, considering that no changes are observed in pure LiClO_4_ (Figure 4a) and that the reconstruction is stable in pure CsClO_4_ (Figure 1c), we have no reason to assume that the reconstruction gets lifted in the electrolyte mixture. Still, to clarify this further, we have added a simulation of the CTR profile for (partially) lifted Au(100) hexagonal reconstruction in the SI (Figure S8b, made using the parameters from Table S1) without a Cs^+^ layer. It can be seen how the lifting affects both the shape and symmetry of the specular CTR very differently than the changes we observe experimentally and assign to the Cs^+^ layer.

**Figure 5:**
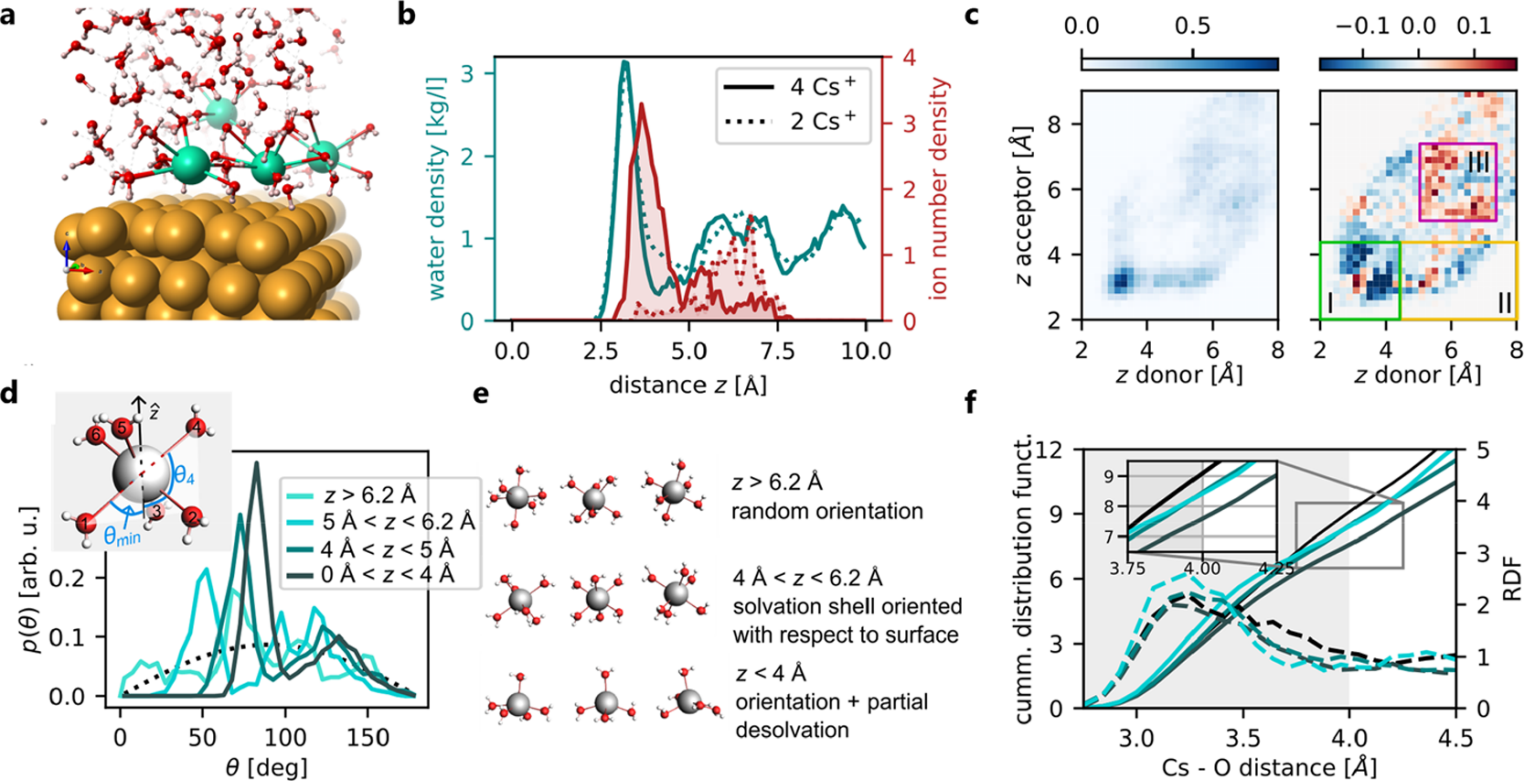
Results from Ab Initio Molecular Dynamic (AIMD) simulations. (**a**) Snapshot of a simulation with 4 ions at the interface. The ions mostly locate close to the interface with a partially open solvation shell. (**b**) Probability distribution of water (teal) and ions (red) above the surface (surface is located at *z* = 0) for the simulation with 4 ions (full line) and 2 ions (dotted line) in the simulation cell. (**c**) Left: probability distribution of finding hydrogen bond donors and acceptors in the simulation with 4 ions; right: difference to the simulation containing 2 ions. Regions I, II, and II: intralayer H-bonding in the first water layer (reduced), interlayer H-bonding between first and second water layer (reduced), and intralayer H-bonding in the second water layer (increased). (**d**) Angle distribution for Cs^+^-O bonds as defined in the inset. The water molecule forming the smallest angle θ defines θ_min_. (**e**) Schematic sketch for the behavior of the ion’s solvation at different ion-surface distances *z*. [Note that we use a Cs^+^—water coordination number of 6 here to simplify the sketch. The true coordination number of Cs^+^ is higher (see panel (f))] (**f**) radial distribution function (RDF; dotted line) and cumulative distribution function (full line) for the Cs^+^-water coordination for ions residing at different heights *z* as specified in panel (d) (data based on the simulation containing 4 ions; data for *z* > 6.2Å not shown—see SI, Section 1.4 for details). Black lines: similar, but for a simulation cell containing a single Cs^+^ ion in bulk water.

We can therefore conclude that the previously observed Cs^+^ layer with a well-defined structure found in pure CsClO_4_ (pH 3) is not present when Cs^+^ is mixed to a Li^+^ background. It must be noted that there must, however, be some degree of ordering, otherwise no change in diffraction intensity would be observed in Figure 4a. Still, the presence of Li^+^ makes the Cs^+^ layer significantly more disordered. This increased disorder manifests as the inability to describe the data with a single Cs^+^ layer characterized by a well-defined average height and Debye–Waller factor, implying a broader distribution of Cs^+^ positions and/or enhanced lateral disorder rather than a discrete, ordered layer. This lack of Cs^+^ layer and consequently larger accessibility of the surface is likely one of the contributors to the differences in HER activity.

### AIMD Simulations of Cs^+^-H_2_O–Au Interactions

From the SXRD experiment, we know that the Cs^+^ in the EDL must be (partially) desolvated, although detailed information about this process cannot be obtained. Therefore, AIMD simulations, in which the dynamics of the ion, the water and the surface layers are considered, were used to analyze the ion’s solvation. Because AIMD simulations are limited in time and size, we do not expect a quantitative agreement with the experimental ion density profiles; instead, we use the simulations to confirm that ions can reach the ion–surface distances observed by SXRD and to analyze the associated solvation structure, which is more reliably captured on the AIMD time scale.

To get as close as possible to the experimental conditions, we consider two situations, which differ in the number of ions (2 and 4 ions) in the simulation cell (see Figure 5a for a snapshot of the 4-ion cell). By tuning the number of ions in the cell, the effective surface charge can be tuned, and the simulations will result in a different electrode potential. For the 2 and 4 ion cases, the potential obtained corresponds to approximately −0.7 V versus Ag/AgCl and −1 V versus Ag/AgCl, similar to the potentials applied in the experiment. The time scales of our AIMD simulations are too short to fully thermalize the Cs^+^-surface distance (see Figure S15). Nevertheless, for the simulation containing 4 ions, which results in a potential of approximately −1.0 V versus Ag/AgCl, we observe a peak of ions residing between 3 Å and 5 Å from the surface, in close quantitative agreement with the experiment (compare Figure 5b,e). Although this agreement may be fortuitous, it allows us to further analyze the ion’s solvation and impact on the hydration structure for realistic ion-surface distances. For the simulation with 2 ions in the simulation cell (resulting in a potential of approximately −0.7 V vs Ag/AgCl), we find the ions to reside considerably further away from the surface. The average distance does not correspond well to the experimental distances found experimentally at −0.50 V versus Ag/AgCl, but the fact that the ions reside (on average) far from the surface allows us to use this simulation as a reference calculation to investigate ion-induced changes at the interface.

As shown in Figure 5b, ions residing between 3 Å and 5 Å from the surface, overlap strongly with the location of the first water layer, located between ∼2.5 Å and ∼4.4 Å. Interestingly, the strong overlap of the ion positions in the simulation with 4 ions with the first water layer results only in relatively small changes in the interfacial water density distribution (compare full and dotted turquoise lines in Figure 5b): the position of the first water layer is not impacted at all by the ions, and the number of water molecules in the first water layer is only reduced by 13% when comparing the simulation with 4 ions to that with 2 Cs^+^ ions (which reside on average much further from the surface in our simulations). The largest changes in the water density occur in the low-density region between the first and the second water layer, where the presence of more ions close to the interface seems to further decrease the water density. Concurrently, the hydrogen bonding network (see SI for definition) from the second water layer to the first water layer is also slightly weakened (see Figure 5c). Additional changes in the hydrogen bonding network occur within the first water layer: in the simulation with 4 ions, the intralayer hydrogen bonding in the first layer, is reduced by nearly 1/3 compared to the case with only 2 ions. This decrease could be related directly to the presence of the (chaotropic) Cs^+^ ions.^48^ It could, however, also be an indirect effect caused by the increased surface charge in the 4 Cs^+^ simulation, which orients the water molecules more strongly H-down. Based on an analysis of snapshots with the same total number of ions, but different numbers of ions close to the interface, we conclude that the actual presence of the (chaotropic) Cs^+^ ions at the interface plays a significant role in reducing the hydrogen bonding network in the first interfacial water layer (see Table S3 and SI Section 1.3). This reduced hydrogen bonding is consistent with experimental observations based on surface-enhanced infrared absorption spectroscopy measurements^10,23^ as well as with previous computational findings.^50^ The reduced hydrogen bonding network within the first layer and from the second to the first layer may impact the hydrogen evolution reaction as a weaker hydrogen bonding network can be expected to increase the barrier for H adsorption (H-bonds may stabilize OH^δ−^-H^δ+^ intermediates). In summary, the simulation suggests that the presence of ions at the interface has a significant impact on the hydrogen bonding, but only a minor impact on the water density distribution directly at the interface.

Turning to the ion and its solvation, Figure 5a shows a typical snapshot obtained during our AIMD simulations. In this snapshot, the four ions reside at 3.6 Å, 4.1 Å, 4.2 Å, and 7.0 Å from the surface. The two ions residing closest to the interface thus represent the ion-surface distance found experimentally at −1.25 V versus Ag/AgCl well. It is clear from the image that these Cs^+^ ions are too close to the surface to allow for any water molecules to be situated between the ion and the surface, leading to a solvation shell that is open toward the surface. As shown in Figure 5d, an (on average) spherically symmetric solvation shell is only found for ions residing more than ∼6Å from the surface. Already for ion—surface distances between 5 Å and 6.2 Å, the averaged solvation shell is clearly nonspherical and, for ions residing between 4 Å and 5 Å from the surface, no water molecules are found within an opening angle of 2θ_min_ = 100° (see Figure 5d). Despite this large opening angle, the coordination number is not necessarily reduced. Instead, to achieve this opening angle, the ions can rotate their solvation shell to optimally align it with the surface as schematically shown in Figure 5e and explained in more detail in the Section 1.5.1 in the SI. A slight distortion in the solvation, allowing more O atoms to reside close to each other, may further facilitate the formation of such a large opening angle (see Figure S14 in the SI). Ions residing even closer to the interface (*z* < 4 Å) are characterized by an even larger opening angle of 2θ_min_ = 130° which is likely accompanied by a ∼10% decrease in the Cs^+^-water coordination (see Figure 5f). Based on a simple energetic analysis presented in the SI, Section 1.5.2, the energetic cost related to the (relatively small) loss in solvation can be readily compensated by a reduction in electrostatic energy stored in the electric double layer due to the ion approaching the surface more closely. This closer approach to the surface is facilitated by the reduced solvation of the ion: As shown in the SI, Section 1.5.1 and as schematically depicted in Figure 5e, the loss of only one water molecule allows the opening angle to increase to 150° without any further distortion of the solvation shell.

Overall, our simulations suggest that the solvation shell of Cs^+^ is initially oriented to optimally arrange the solvating water molecules with respect to the surface, and slightly distorted as the ion approaches the surface. This situation should hold true for ion-surface distances as found experimentally at −0.15 V versus Ag/AgCl at pH = 13. As the ions approach the interface even closer (as experimentally found for lower potentials), the Cs^+^ ions partially shed their solvation, with the energetic cost likely being compensated by a gain in electrostatic stability caused by the ion moving closer to the charged interface. Independently of the potential, the ion-surface distances found experimentally at the potentials investigated are so short that no water molecules can reside directly between the ion and the surface, contradicting Cs^+^ geometries at the electrochemical interface proposed previously.^5,51,52^

## Discussion

Figure 6 shows a schematic representation of the double layer structure measured using SXRD under different experimental conditions and further elucidated in the simulations. When the potential is made more negative, Cs^+^ cations accumulate near the surface, that is, reach a higher coverage, but, importantly, they also gradually move closer to the surface. In the latter process, the solvation shell is first distorted, while at the most negative potential, part of the solvation shell is shed. This picture adds detail to the classic model of outer- and inner-Helmholtz plane, and indicates these are not fixed planes, but they change and transform into each other as a function of potential. Such a dense and ordered Cs^+^ layer is no longer detected when Cs^+^ ions are mixed with Li^+^ (Figure 4). Still, a more disordered Cs^+^ layer preferentially occupies the double-layer despite the presence of enough Li^+^ to screen the surface charge, likely due to the weak interaction between Cs^+^ and water molecules, and consequently higher mobility and interaction with the surface. We point out that even though Li^+^ is known to possess a rigid and strongly bound first hydration shell in aqueous solution, simulations indicate that partial distortion or dehydration of Li^+^ can occur near highly charged metal-water interfaces or under strong interfacial electric fields, although such effects are expected to be much less pronounced than for larger, weakly hydrated alkali cations such as Cs^+^.

**Figure 6:**
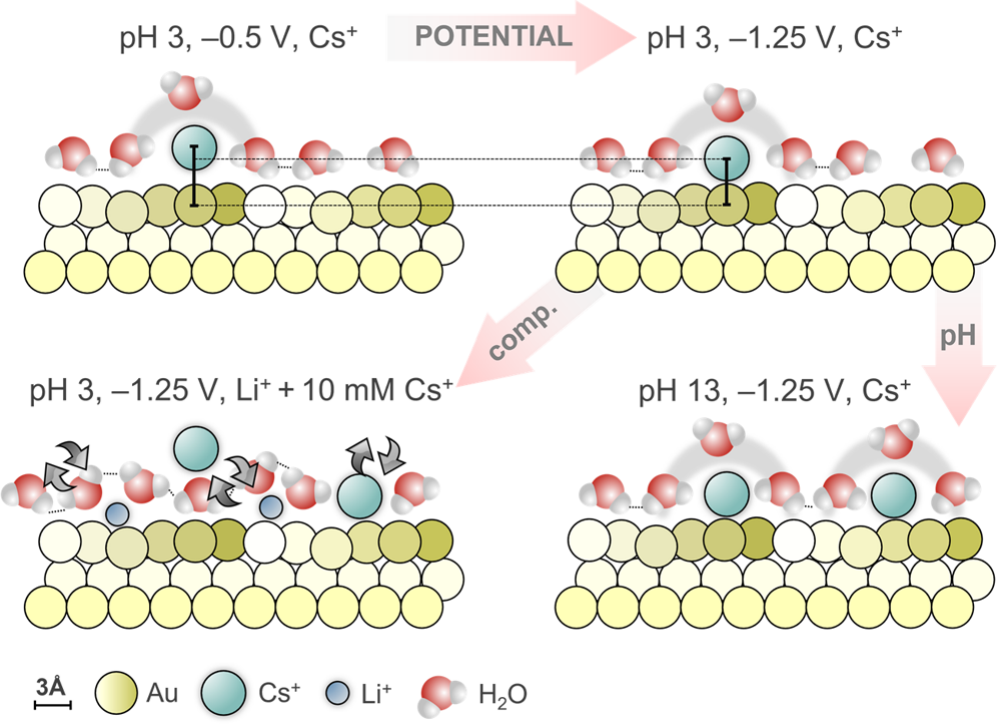
Schematic representation of the two-dimensional structure of the double-layer. Effect of potential, pH and electrolyte composition, based on the SXRD and AIMD data. All atoms and cation-to-surface distances are drawn to scale. Gold atoms are depicted in shades of yellow, Cs^+^ ions are represented in green, Li^+^ ions in blue, and water molecules in red (O) and white (H). The arrows indicate the effect of the different variables studied on the surface–cation interaction.

In bulk electrolyte solutions, in which cations and anions reside relatively far apart on average, these species can already affect the structure of water by disturbing the extent of hydrogen bonding and consequently mobility of water molecules. Chaotropic ions (like Cs^+^), also called “structure breakers” disrupt the hydrogen bonding network between water molecules more easily while kosmotropic species (e.g., Li^+^), promote or enhance the degree of H-bonding in bulk water.^48^ These tendencies can be quantitatively measured and are described by the “Jones–Dole β-coefficient” described by Marcus,^48^ which is a function of the conductivity, viscosity and concentration of the salt, pertaining to infinite dilution under the common assumption that [anion] = [cation]. The underlying thermodynamic component of this behavior is correlated to the iońs entropy of hydration, and the different properties of water molecules confined on the surrounding of a cation. In this context, Li^+^ forms a rigid and strongly oriented hydration shell in which water molecules are tightly bound with hydrogen atoms preferentially pointing outward, whereas the more weakly hydrated Cs^+^ ion supports a looser solvation structure that can retain internal hydrogen bonding between water molecules and exhibits a less constrained orientational ordering. The SXRD results from Figure 4 demonstrate that when it comes to electrolyte mixtures, the simple “structure-making/breaking” might not be enough to explain interfacial properties, as we find that the double layer in such mixture is likely populated by a distribution of both Li^+^ and Cs^+^ ions which does not follow bulk ratios, with the ions likely at different distances from the electrode surface, and different degrees of mobility and interaction with water.

On gold, the rate-determining step for water reduction (in neutral and alkaline media) is the first electron transfer, corresponding to the dissociation of water (Volmer step, eq 1), while the dissociation of H_3_O^+^ (in acidic media) is much faster. Alkali metal cations near the surface are proposed to stabilize the transition state of water dissociation near the surface at low cation concentrations (eq 2),^8,24,44^ while according to our previous results they do not strongly influence proton reduction on gold.

1 $$H_{2}O+e^{-}+*\to H-*+{OH}^{-}$$

2 $$H_{2}O+e^{-}+*+M^{+}\to *H-{OH}^{\delta -}{-M}^{+}+{\left(1-\delta \right)e}^{-}\to *H+{OH}^{-}+M^{+}$$

Based on the mechanisms shown in eqs 1,2, there are, in principle, three main ways by which the higher Cs^+^ coverages found in our study can influence the activity of the water reduction reaction on gold. First, based on our AIMD simulations, the hydrogen bonding network is disrupted in the presence of ions close to the surface. This can affect the Volmer barrier or the barrier to transport the OH^–^ species away from the surface. Second, the water density in the first water density minimum is further decreased in the presence of ions, which may simply lower the number of reactants. Third, for ions residing close to the interface (as may be more likely at low potentials, but potentially also at high concentrations), the water molecules reside less and less in between the ions and the surface, such that they should be exposed to a decreased electric field. We do not intend, however, to establish unequivocally which cation effects are or are not in play during alkaline HER. On the contrary, based on our results and previous literature data, there is likely no “one-model-fits-all”. Our findings from SXRD and AIMD, summarized schematically in Figure 6, rather suggest another mechanism that can also be at play that has not found much attention so far, namely the ordering of the cation layer itself having an effect on the accessibility of the surface sites and on the overall double-layer dynamics.

## Conclusions

Through a combined analysis of the specular CTR results and AIMD, we can draw a more data-based picture of the atomic structure at the electrified interface during HER on gold at different applied potentials, pH and electrolyte composition. We observe that Cs^+^ cations have well-defined out-of-plane positions in pure Cs^+^-electrolytes, with higher coverages found at higher pH. Our findings underscore the significance of considering the influence of cation structure and associated double-layer ordering on the hydrogen evolution and other electrocatalytic reactions, aside from the already known cation effects on water dissociation, OH adsorption and water H-bonding network. While SXRD experiments under electrochemical conditions have previously provided valuable insights into the interface structure primarily as a function of the potential, they often have limited correlation to electrocatalysis. In the future more detailed SXRD studies can further aid in characterizing how electrolyte species interact with electrode surfaces, and especially on how to transform these insights into improvements in electrocatalytic activity.

## Supplementary Material



## Notes

Supporting discussion to experimental and theoretical data, Au(100) electrode characterization, additional SXRD measurements, parameters for CTR fits and simulations, details on AIMD simulations, Supporting Methods (PDF)

## References

[1] Wang X., Ruan Q., Sun Z. (2023). Minireview of the Electrocatalytic
Local Environment in Alkaline Hydrogen Evolution. Energy Fuels.

[2] Moura
de Salles Pupo M., Kortlever R. (2019). Electrolyte Effects on the Electrochemical
Reduction of CO_2_. ChemPhysChem.

[3] Xu P., Wang R., Zhang H., Carnevale V., Borguet E., Suntivich J. (2024). Cation Modifies
Interfacial Water
Structures on Platinum during Alkaline Hydrogen Electrocatalysis. J. Am. Chem. Soc..

[4] Khani H., Puente Santiago A. R., He T. (2023). An Interfacial View of Cation Effects
on Electrocatalysis Systems. Angew. Chem. Int.
Ed..

[5] Shah A. H., Zhang Z., Huang Z., Wang S., Zhong G., Wan C., Alexandrova A. N., Huang Y., Duan X. (2022). The Role of
Alkali Metal Cations and Platinum-Surface Hydroxyl in the Alkaline
Hydrogen Evolution Reaction. Nat. Catal..

[6] Ding X., Garlyyev B., Watzele S. A., Kobina Sarpey T., Bandarenka A. S. (2021). Spotlight on the Effect of Electrolyte
Composition
on the Potential of Maximum Entropy: Supporting Electrolytes Are Not
Always Inert. Chem. Eur. J..

[7] Sebastián-Pascual P., Shao-Horn Y., Escudero-Escribano M. (2022). Toward Understanding the Role of
the Electric Double Layer Structure and Electrolyte Effects on Well-Defined
Interfaces for Electrocatalysis. Curr. Opin.
Electrochem..

[8] Bender J. T., Petersen A. S., Østergaard F. C., Wood M. A., Heffernan S. M. J., Milliron D. J., Rossmeisl J., Resasco J. (2023). Understanding Cation
Effects on the Hydrogen Evolution Reaction. ACS Energy Lett..

[9] Huang B., Muy S., Feng S., Katayama Y., Lu Y. C., Chen G., Shao-Horn Y. (2018). Non-Covalent
Interactions in Electrochemical Reactions
and Implications in Clean Energy Applications. Phys. Chem. Chem. Phys..

[10] Huang B., Myint K. H., Wang Y., Zhang Y., Rao R. R., Sun J., Muy S., Katayama Y., Corchado Garcia J., Fraggedakis D., Grossman J. C., Bazant M. Z., Xu K., Willard A. P., Shao-Horn Y. (2021). Cation-Dependent Interfacial Structures
and Kinetics for Outer-Sphere Electron-Transfer Reactions. J. Phys. Chem. C.

[11] Monteiro M. C. O., Goyal A., Moerland P., Koper M. T. M. (2021). Understanding
Cation Trends for Hydrogen Evolution on Platinum and Gold Electrodes
in Alkaline Media. ACS Catal..

[12] Goyal A., Louisia S., Moerland P., Koper M. T. M. (2024). Cooperative Effect
of Cations and Catalyst Structure in Tuning Alkaline Hydrogen Evolution
on Pt Electrodes. J. Am. Chem. Soc..

[13] Ojha K., Arulmozhi N., Aranzales D., Koper M. T. M. (2020). Double Layer
at the Pt(111)–Aqueous Electrolyte Interface: Potential of
Zero Charge and Anomalous Gouy–Chapman Screening. Angew. Chem., Int. Ed..

[14] Ojha K., Doblhoff-Dier K., Koper M. T. M. (2022). Double-Layer Structure of the Pt(111)–Aqueous
Electrolyte Interface. Proc. Natl. Acad. Sci.
U. S. A..

[15] Doblhoff-Dier K., Koper M. T. M. (2023). Electric Double Layer of Pt(111): Known Unknowns and
Unknown Knowns. Curr. Opin. Electrochem..

[16] Herasymenko P., Šlendyk I. (1930). Wasserstoffüberspannung Und
Adsorption Der Ionen
[Hydrogen Evolution Overpotential and Adsorption of Ions]. Z. Phys. Chem. A.

[17] Waegele M. M., Gunathunge C. M., Li J., Li X. (2019). How Cations Affect
the Electric Double Layer and the Rates and Selectivity of Electrocatalytic
Processes. J. Chem. Phys..

[18] Ovalle V.
J., Hsu Y.-S., Agrawal N., Janik M. J., Waegele M. M. (2022). Correlating
Hydration Free Energy and Specific Adsorption of Alkali Metal Cations
during CO2 Electroreduction on Au. Nat. Catal..

[19] Frumkin A. N. (1959). Influence
of Cation Adsorption on the Kinetics of Electrode Processes. Trans. Faraday Soc..

[20] Dunwell M., Wang J., Yan Y., Xu B. (2017). Surface Enhanced Spectroscopic
Investigations of Adsorption of Cations on Electrochemical Interfaces. Phys. Chem. Chem. Phys..

[21] Chen X., McCrum I. T., Schwarz K. A., Janik M. J., Koper M. T. M. (2017). Co-Adsorption
of Cations as the Cause of the Apparent PH Dependence of Hydrogen
Adsorption on a Stepped Platinum Single-Crystal Electrode. Angew. Chem. Int. Ed..

[22] Jiao L., Liu E., Mukerjee S., Jia Q. (2020). In Situ Identification of Non-Specific
Adsorption of Alkali Metal Cations on Pt Surfaces and Their Catalytic
Roles in Alkaline Solutions. ACS Catal..

[23] Huang B., Rao R. R., You S., Hpone Myint K., Song Y., Wang Y., Ding W., Giordano L., Zhang Y., Wang T., Muy S., Katayama Y., Grossman J. C., Willard A. P., Xu K., Jiang Y., Shao-Horn Y. (2021). Cation- and PH-Dependent Hydrogen
Evolution and Oxidation
Reaction Kinetics. JACS Au.

[24] Goyal A., Koper M. T. M. (2021). The Interrelated Effect of Cations
and Electrolyte
PH on the Hydrogen Evolution Reaction on Gold Electrodes in Alkaline
Media. Angew. Chem., Int. Ed..

[25] Goyal A., Koper M. T. M. (2021). Understanding
the Role of Mass Transport in Tuning
the Hydrogen Evolution Kinetics on Gold in Alkaline Media. J. Chem. Phys..

[26] Harlow G.
S., Lundgren E., Escudero-Escribano M. (2020). Recent Advances in Surface X-Ray
Diffraction and the Potential for Determining Structure-Sensitivity
Relations in Single-Crystal Electrocatalysis. Curr. Opin. Electrochem..

[27] Gründer Y., Lucas C. A. (2016). Surface X-Ray Diffraction
Studies of Single Crystal
Electrocatalysts. Nano Energy.

[28] Nakamura M., Nakajima Y., Hoshi N., Tajiri H., Sakata O. (2013). Effect of
Non-Specifically Adsorbed Ions on the Surface Oxidation of Pt(111). ChemPhysChem.

[29] Liu Y., Kawaguchi T., Pierce M. S., Komanicky V., You H. (2018). Layering and Ordering in Electrochemical Double Layers. J. Phys. Chem. Lett..

[30] Nakamura M., Nakajima Y., Kato K., Sakata O., Hoshi N. (2015). Surface Oxidation
of Au(111) Electrode in Alkaline Media Studied by Using X-Ray Diffraction
and Infrared Spectroscopy: Effect of Alkali Metal Cation on the Alcohol
Oxidation Reactions. J. Phys. Chem. C.

[31] Wang J., Ocko B. M., Davenport A. J., Isaacs H. S. (1992). In Situ X-Ray-Diffraction
and Reflectivity Studies of the Au(111)/Electrolyte Interface: Reconstruction
and Anion Adsorption. Phys. Rev. B.

[32] Tanaka S., Tajiri H., Sakata O., Hoshi N., Nakamura M. (2022). Interfacial
Structure of Pt(110) Electrode during Hydrogen Evolution Reaction
in Alkaline Solutions. J. Phys. Chem. Lett..

[33] Kawaguchi T., Liu Y., Karapetrova E. A., Komanicky V., You H. (2020). In-Situ to Ex-Situ in-Plane Structure
Evolution of Stern Layers on
Pt(111) Surface: Surface X-Ray Scattering Studies. J. Electroanal. Chem..

[34] Strmcnik D., Van Der Vliet D. F., Chang K. C., Komanicky V., Kodama K., You H., Stamenkovic V. R., Marković N. M. (2011). Effects of Li+, K+, and Ba2+ Cations
on the ORR at
Model and High Surface Area Pt and Au Surfaces in Alkaline Solutions. J. Phys. Chem. Lett..

[35] Nakamura M., Sato N., Hoshi N., Sakata O. (2011). Outer Helmholtz Plane
of the Electrical Double Layer Formed at the Solid Electrode-Liquid
Interface. ChemPhysChem.

[36] Tidswell I. M., Marković N. M., Lucas C. A., Ross P. N. (1993). In Situ
X-Ray-Scattering
Study of the Au(001) Reconstruction in Alkaline and Acidic Electrolytes. Phys. Rev. B.

[37] Ocko B. M., Wang J., Davenport A., Isaacs H. (1990). In Situ X-Ray Reflectivity
and Diffraction Studies of the Au(001) Reconstruction in an Electrochemical
Cell. Phys. Rev. Lett..

[38] Vlieg E. (1997). Integrated
Intensities Using a Six-Circle Surface X-Ray Diffractometer. J. Appl. Crystallogr..

[39] Vlieg E. (2000). ROD: A Program
for Surface X-Ray Crystallography. J. Appl.
Crystallogr..

[40] Jacob T. (2007). Potential-Induced
Lifting of the Au(100)-Surface Reconstruction Studied with DFT. Electrochim. Acta.

[41] Strbac S., Hamelin A., Adzić R. R. (1993). Electrochemical
Indication of Surface
Reconstruction of (100), (311) and (111) Gold Faces in Alkaline Solutions. J. Electroanal. Chem..

[42] Peng L.-M., Ren G., Dudarev S. L., Whelan M. J. (1996). Debye–Waller
Factors and Absorptive
Scattering Factors of Elemental Crystals. Acta
Crystallogr. A.

[43] Monteiro M. C. O., Liu X., Hagedoorn B. J. L., Snabilié D. D., Koper M. T. M. (2022). Interfacial pH
Measurements Using a Rotating Ring-Disc
Electrode with a Voltammetric pH Sensor. ChemElectroChem.

[44] Monteiro M. C. O., Dattila F., López N., Koper M. T. M. (2022). The Role of Cation
Acidity on the Competition between Hydrogen Evolution and CO_2_ Reduction on Gold Electrodes. J. Am. Chem.
Soc..

[45] Marcus Y. (1988). Ionic Radii
in Aqueous Solutions. Chem. Rev..

[46] Monteiro M. C. O., Dattila F., Hagedoorn B., García-Muelas R., López N., Koper M. T. M. (2021). Absence of CO_2_ Electroreduction
on Copper, Gold and Silver Electrodes without Metal Cations in Solution. Nat. Catal..

[47] Banerjee S., Zhang Z., Hall A. S., Thoi V. S. (2020). Surfactant
Perturbation
of Cation Interactions at the Electrode–Electrolyte Interface
in Carbon Dioxide Reduction. ACS Catal..

[48] Marcus Y. (2009). Effect of
Ions on the Structure of Water: Structure Making and Breaking. Chem. Rev..

[49] Alfarano S. R., Pezzotti S., Stein C. J., Lin Z., Sebastiani F., Funke S., Hoberg C., Kolling I., Ma C. Y., Mauelshagen K., Ockelmann T., Schwaab G., Fu L., Brubach J. B., Roy P., Head-Gordon M., Tschulik K., Gaigeot M. P., Havenith M. (2021). Stripping
Away Ion
Hydration Shells in Electrical Double-Layer Formation: Water Networks
Matter. Proc. Natl. Acad. Sci. U. S. A..

[50] Zhang Z., Li H., Shao Y., Gan L., Kang F., Duan W., Hansen H. A., Li J. (2024). Molecular
Understanding of the Critical
Role of Alkali Metal Cations in Initiating CO_2_ Electroreduction
on Cu(100) Surface. Nat. Commun..

[51] Strmcnik D., Kodama K., Van Der Vliet D., Greeley J., Stamenkovic V. R., Marković N. M. (2009). The Role
of Non-Covalent Interactions in Electrocatalytic
Fuel-Cell Reactions on Platinum. Nat. Chem..

[52] Rebstock J. A., Zhu Q., Baker L. R. (2022). Comparing
Interfacial Cation Hydration at Catalytic
Active Sites and Spectator Sites on Gold Electrodes: Understanding
Structure Sensitive CO_2_ Reduction Kinetics. Chem. Sci..

