# Supplementary material for: Cation-Surface Interactions During Electrocatalytic Hydrogen Evolution Probed by Surface X‑ray Diffraction
Source: ACS Phys Chem Au. 2026 Mar 3;6(3):503–13. doi: 10.1021/acsphyschemau.5c00152 (PMC13220183; doi:10.1021/acsphyschemau.5c00152)
Supplement: Supplementary file 1 [file pg5c00152_si_001.pdf]

Cation-surface interactions during electrocatalytic hydrogen evolution probed by Surface X-ray Diffraction

Mariana C. O. Monteiro[a,b,*], Leon Jacobse[a,c], Arthur M. V. Hagopian[b], Vedran Vonk[c], Simon Chung[c,d], Sheena Louisia[b], Alexander Meinhardt[c], Elif Öykü Alagöz[a], Xin Deng[c], Beatriz Roldan Cuenya[a], Katharina Doblhoff-Dier[b,*], Marc T. M. Koper[b], Andreas Stierle[c,e]

[a] *Department of Interface Science, Fritz-Haber Institute of the Max Planck Society, Faradayweg 4-6, 14195 Berlin, Germany*
[b] *Leiden Institute of Chemistry, Leiden University, P.O. Box 9502, 2300 RA Leiden, The Netherlands*
[c] *Centre for X-ray and Nano Science CXNS, Deutsches Elektronen-Synchrotron DESY, Notkestrasse 85, D-22607 Hamburg, Germany*
[d] *Electrical and Computer Engineering, Rice University, 6100 Main Street, Houston, TX 77005, United States*
[e] *Fachbereich Physik, Universität Hamburg, Jungiusstraße 11, 20355 Hamburg, Germany*

***Corresponding author information**
monteiro@fhi-berlin.mpg.de; k.doblhoff-dier@lic.leidenuniv.nl

Supporting Information

1. Supporting Discussion

1.1 *hex*-Au(100) characterization

The in-plane signal corresponding to the Au(100) reconstructed surface rod was recorded before (in an argon atmosphere) contact with the electrolyte, in close agreement with the data from Ocko et al.[1]

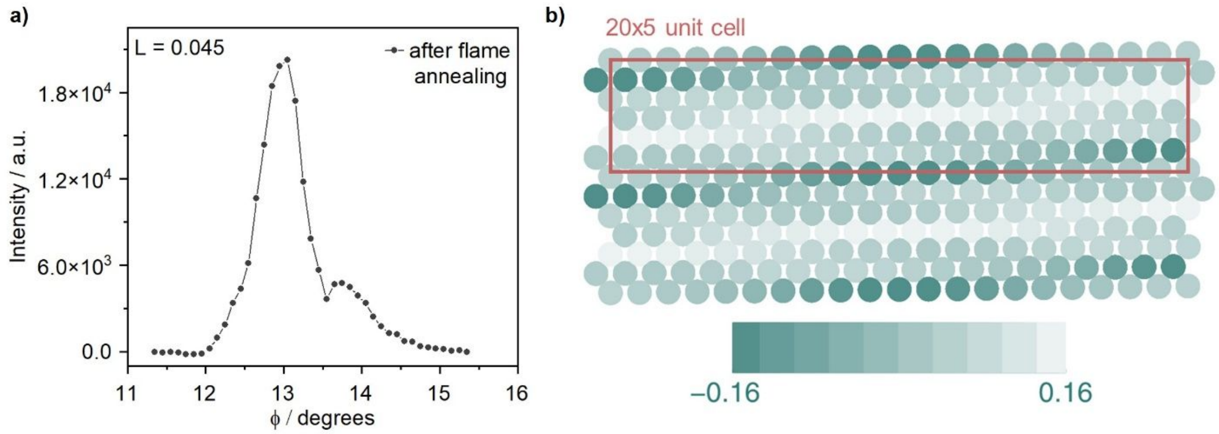

Figure S1. a) In-plane diffraction of the Au(100) hexagonal reconstruction surface before contact with the electrolyte (in an argon atmosphere. **b)** Top view of the hexagonal layer of a Au(100)-5x20 reconstruction model, adapted from Havu et al.[2] The out-of-plane (z) coordinates of all atoms are in different green scales in units of the bulk interlayer spacing, relative to the average z position of the plane.

The blank voltammetry of the hex-Au(100) electrode in the SXRD cell after flame annealing shows distinct features between –0.1 and 0.2 V vs. Ag/AgCl. The anodic peaks are associated with the potential-induced lifting of the 20x√5 hexagonal layer, forming a 1x1 structure. Because the point of zero charge of the unreconstructed 1x1-Au(100) surface is more negative than of the *hex*-Au(100), as the potential is scanned positively and the reconstruction lifts, the surface charge becomes more positive leading to an increase in the double-layer charging current as anions in the electrolyte suddenly move towards the electrode surface.

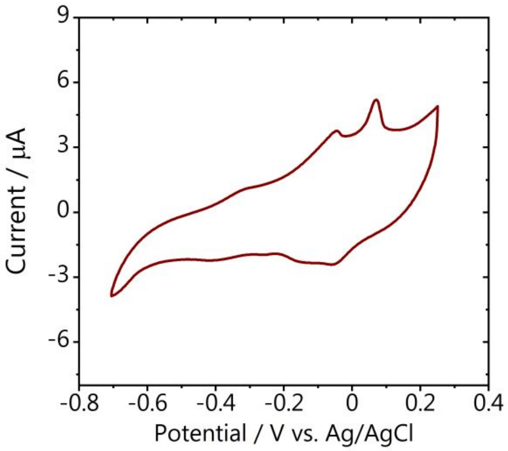

Figure S2. Blank voltammetry of the as-prepared *hex*-Au(100) electrode in the electrochemical cell mounted in the SXRD setup, recorded at 2 mV s^{-1} in 0.1 M CsOH (pH 13).

Figure S3 contains radial in-plane scans of the *hex*-Au(100) electrode in the [1 1 0] direction recorded at L = 0.045 at a constant potential (–0.5 V vs. Ag/AgCl) before and after the specular CTR measurements from Figure 1 in the main text, and cyclic voltammetry. Electrochemical cycling is known to be a way of recovering the hexagonal layer, however we see that after 10 cycles at 10 mV s^{-1} this only partially occurs. In fact, the chronoamperometric data (Figure S4b) indicates that the kinetics of lifting the reconstruction electrochemically are relatively fast at –0.1 V vs. Ag/AgCl, compared to the kinetics to form it again by cycling (Figure S3b). Importantly, only looking at the *hex*-Au(100) blank voltammetry (Figure S2), one would say that the hexagonally reconstructed surface is stable at –0.1 V vs. Ag/AgCl, and literature often assigns that the "lifting" of the reconstruction takes place mainly beyond 0.1 V vs. Ag/AgCl. Nonetheless, we see here that the reconstruction is already affected at potentials more negative than expected.

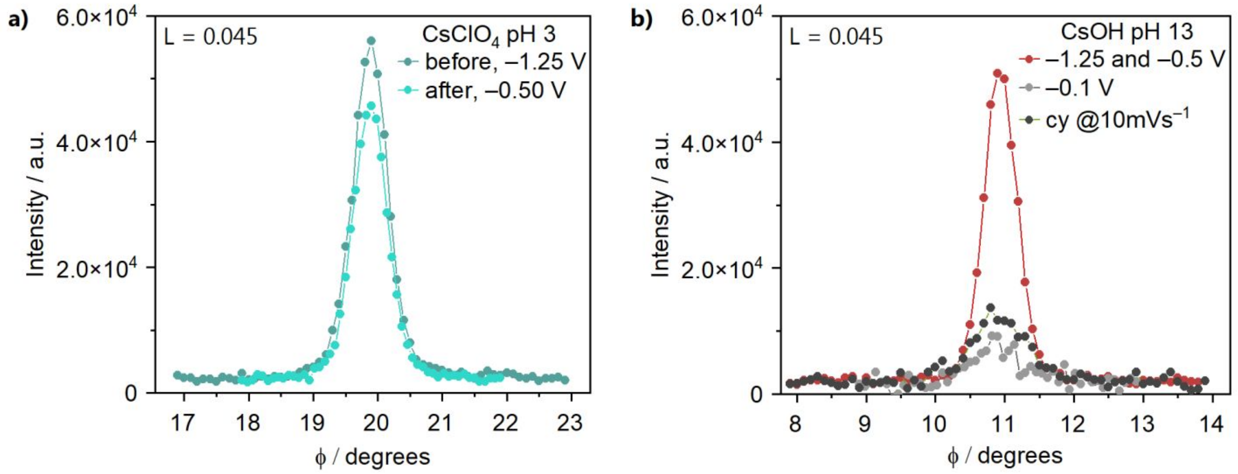

Figure S3. In-plane diffraction of the Au(100) hexagonal reconstruction surface **a)** before and after the specular CTR measurements at pH 3, b) after the specular CTR measurements at pH 13 at –0.5 and –1.25 V (red); at –0.1 V (gray); and after 10 cycles between –0.5 and –1.25 V at 10 mV s^{-1} as an attempt to reform the hexagonal reconstruction layer. Potentials are reported versus Ag/AgCl.

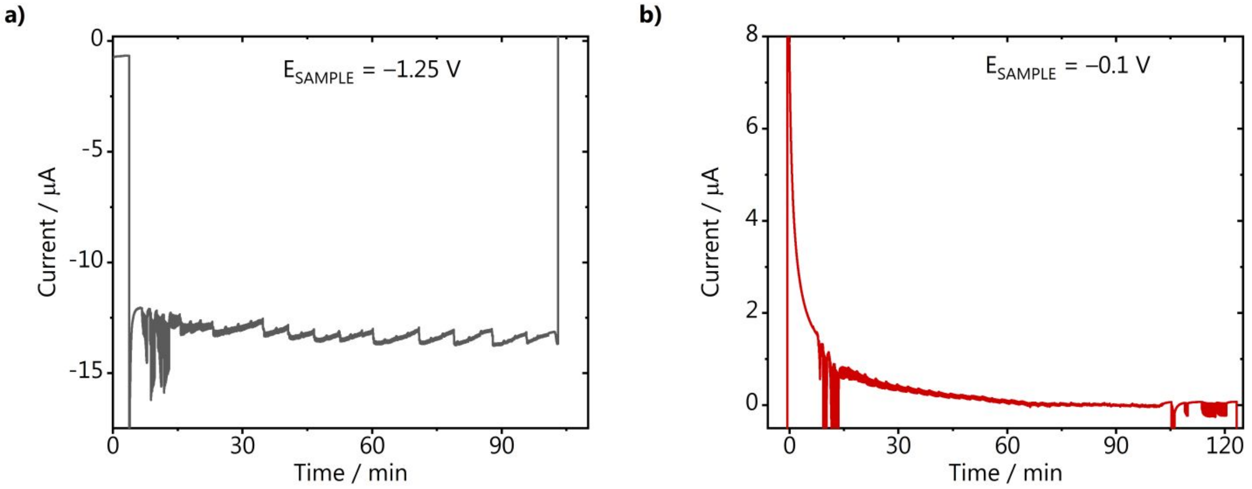

Figure S4. Current recorded during chronoamperometry at a) –1.25 V (hydrogen evolution) and b) –0.1 V (lifting of the reconstruction), during the specular CTR measurement at pH 13. The staircase profile from Figure a) is due to refilling of the meniscus. Potentials are versus Ag/AgCl.

1.2 CTR fits and simulations

Table S1. Parameters used for the simulation from Figure S4. Here, "d" represents the displacement, "Occ" the occupancy, and "DW" the anisotropic root mean square displacement (static Debye-Waller factor) for the different layers from Figure S3. Parameters related to the Au layers were taken from Ocko et al.[1] and Peng et al.[3]. Parameters for the Cs layer were chosen within ranges previously calculated or reported from SXRD measurements in different conditions than in this study.

Parameter	value
**d Cs^+-Au_{hex} / Å**	3.5-4.0
**Δd Au_{hex} -Au_2 / Å**	0.1
**Occ Cs^+ / ML**	1-1.30
**Occ Au_{hex} / ML**	1.20
**Occ Au_2 / ML**	1.0
**DW Cs^+ / $Å^2$**	15
**DW Au_{hex} / $Å^2$**	10
**DW Au_2 / $Å^2$**	2.02
**DW Au_1 / $Å^2$**	0.64
**DW Au_{bulk} / $Å^2$**	0.63

Table S2. Parameters obtained from the fits shown in Figure 2 in the main text. Here, "d" represents the displacement, "Occ" the occupancy, and "DW" the anisotropic root mean square displacement (static Debye-Waller factor) for the different layers from the model shown in the main text, Figure 2c.

Parameter	pH 13						pH 3			
	**-0.1**	**error**	**-0.5**	**error**	**-1.25**	**error**	**-0.5**	**error**	**-1.25**	**error**
**d Cs^+-Au_{hex} / Å**	4.65	0.04	3.87	0.02	3.71	0.01	3.96	0.02	3.74	0.01
**d Au_{hex}–Au_2 / Å**	2.32	0.20	2.46	0.26	2.41	0.07	2.44	0.25	2.473	0.20
**DW Au_{bulk} / $Å^2$**	0.63	-	0.63	-	0.63	-	0.63	-	0.63	-
**DW Au_1/ $Å^2$**	0.64	-	0.64	-	0.64	-	0.64	-	0.64	-
**DW Au_2/ $Å^2$**	3.00	0.35	1.98	0.62	2.02	0.45	2.12	0.62	2.12	0.55
**DW Au_{hex} / $Å^2$**	21.82	2.87	6.29	0.09	8.54	1.07	5.67	0.83	6.65	1.26
**DW Cs^+/ $Å^2$**	13.54	1.17	15.00	1.19	14.99	2.01	15.00*	-	15.00*	-
**Occ Au_2 / ML**	1	-	1	-	1	-	1	-	1	-
**Occ Au_{hex} / ML**	1.13	0.06	1.21	0.05	1.22	0.04	1.17	0.05	1.20	0.04
**Occ Cs^+ / ML**	0.15	0.06	0.16	0.06	0.22	0.04	0.16	0.06	0.18	0.05
**norm Chi²**	1.10	-	1.33	-	1.04	-	1.20	-	1.36	-

*DW Cs^+ was fixed at pH 3, to the same average value found from the pH 13 measurement fits, to make sure differences in occupancy are comparable between both conditions, considering the similar effect changes in Occ and DW cause to the CTR signal intensity.

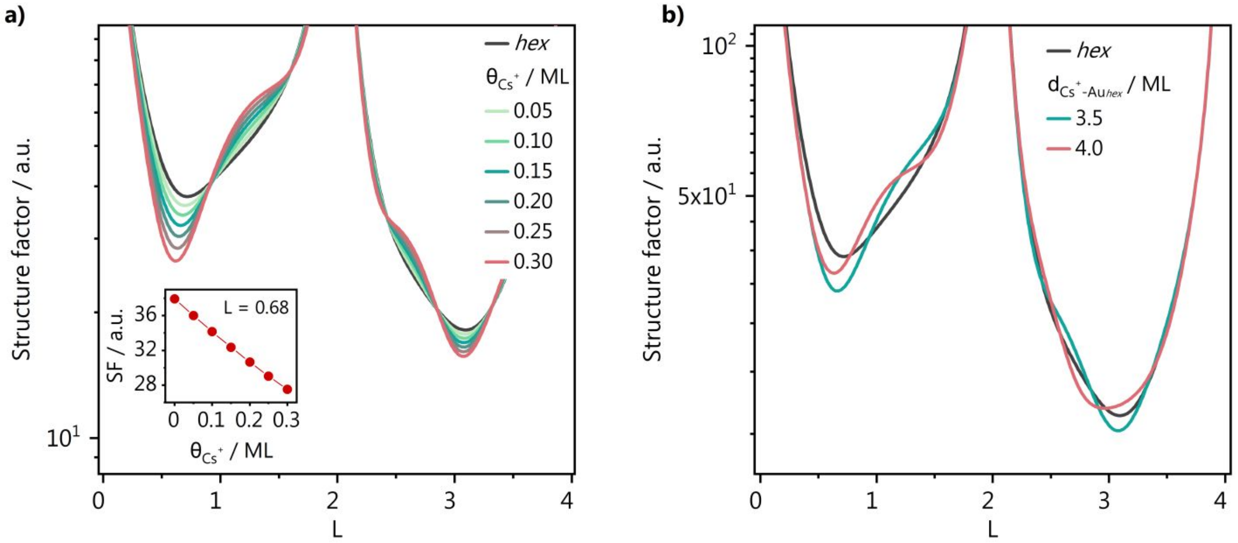

Figure S5. Simulated specular CTRs for the hexagonally terminated Au(100) surface **a)** with different coverages of Cs^+ located 3.5 Å from the Au_{hex} layer and **b)** with Cs^+ at different distances from the surface at a constant coverage of 0.15 ML. The inset from a) shows the signal intensity as a function of Cs^+ coverage at L = 0.6.

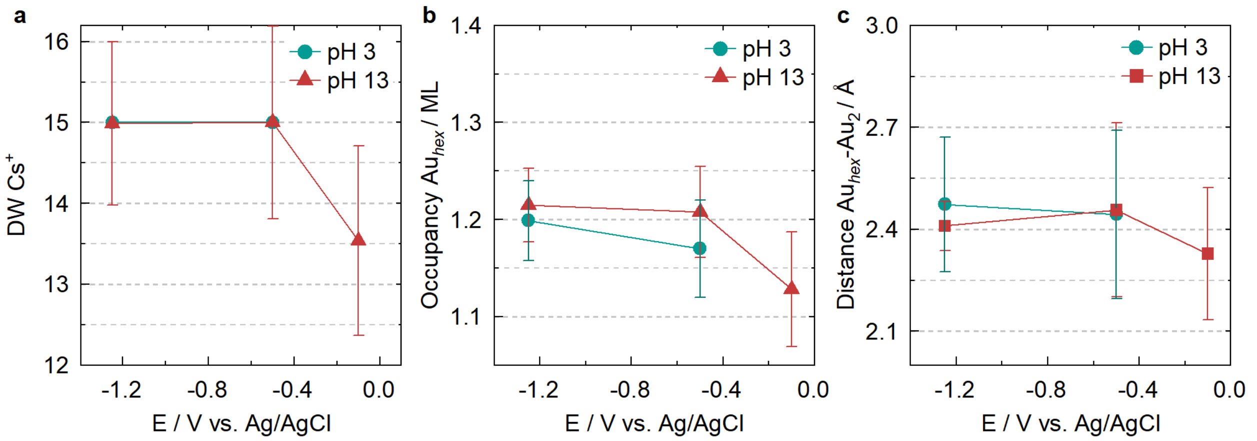

Figure S6. Fit results: **a)** Debye-Waller factors of the Cs^+ layer, **b)** occupancy and **c)** displacement of the Au*hex* layer.

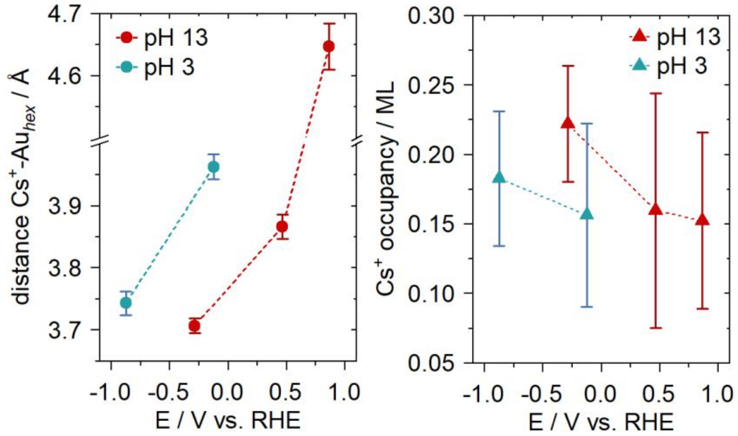

Figure S7. Cs^+ occupancy and Cs^+-surface distance at different pH with potentials plotted versus the reversible hydrogen scale (RHE).

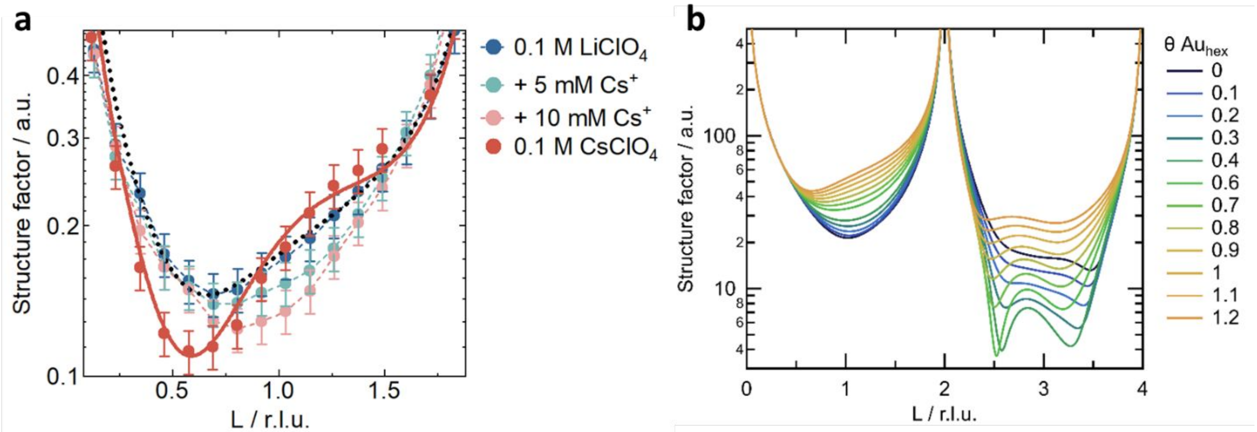

Figure S8. Comparison of specular CTRs recorded at –1.25 V vs. Ag/AgCl in pure 0.1 M $LiClO_4$, 0.08 M $CsClO_4$, and 0.1 M $LiClO_4$ with the addition of 5 and 10 mM of $CsClO_4$. The electrolyte pH was kept constant (pH = 3) by the addition of 1 mM $HClO_4$. The black dotted line is the simulated specular CTR of a "bare" *hex*-Au(100) surface (without a Cs layer), obtained using the parameters from Table S1 and the structural model shown in Figure 2a in the main text.

1.3 Hydrogen bonding network

In the following, we analyze the influence of the exact ion position on the changes in the hydrogen bonding network as discussed in the main text. To this end, we split the trajectory containing 4 ions into snapshots in which all 4 ions reside below $z = 4.7$ Å, snapshots in which a maximum of 2 ions reside below $z = 4.7$ and the rest. For each of the cases, as well as for the total trajectories with 4 and 2 ions, we compute the total number of hydrogen donors residing at $z < 4.4$ Å from the surface. The results are compiled in Table S3 and indicate that the presence of the ions (and not only the overall surface charge) has an important influence on the hydrogen bonding network in (or to be precise from) the first layer. The surface charge may also play a role though.

Table S3: Analysis of the number of H donors residing below $z = 4.4$ Å from the surface.

	**#H-donors at z < 4.4 Å**	**Relative change compared to 2 ion case**
**4 ions (total)**	20	–33%
**4 ions, all of which @ $z < 4.7$ Å**	19	–35%
**4 ions, <2 of which @ $z < 4.7$ Å**	22	–23%
**2 ions**	29	-

1.4 Coordination number and statistical reliability

Due to the shape of the radial distribution function (which does not show a clear minimum after the first solvation sphere), the coordination number cannot be unambiguously determined from the simulations as the coordination number extracted depends sensitively on the chosen cutoff-radius. We use here a cutoff radius of 3.999 Å. Increasing this cutoff radius to 4.2 Å would increase the coordination number for ions found in the bulk region by about 15%, decreasing the cutoff radius to 3.8 Å, would decrease the coordination number of ions found in the bulk region by approximately the same amount.

The coordination number is also subject to statistical fluctuations. Figure S10 shows the change in coordination number of the 4 ions present in the simulation cell over time. The coordination number varies strongly, but there is strong serial correlation visible on the timescales of the AIMD simulation performed. This limits the statistical certainty of the coordination number extracted. In particular, we also find the coordination number for ions in the bulk region ($z > 6.2$ Å) in the simulation with 4 ions in the simulation cell to differ from that with 2 ions in the simulation cell, as well as from a simulations with a single ion (and no electrode) in a 3D-periodic $15x15x15$ Å^3 simulation cell. As shown in Table S4, the coordination

numbers for these 3 cases are 7.8, 8.9 and 8.8, respectively, suggesting an error bar of approximately ± 1 (or 13%) in the coordination number when using a cutoff radius of 3.999 Å.

One should note, however, that in the 4-ion simulation (which differs most strongly from the others) the statistical uncertainty is also highest in the bulk region, as the bulk region is only populated during 5.2 ps (see Table S4). Nevertheless, it shows the statistical uncertainty in our simulations and explains why the simulations do not allow for a statement whether or not the ion may slightly desolvate when moving from the bulk to a position up to $z = 5$ Å from the surface.

A better statistical significance, however, can be expected when analyzing changes in the coordination number for ions going from region 2 (4 Å $< z <$ 5 Å from the surface) to region 1 ($z <$ 4 Å), as two of the ions (ions number 2 and 4) rapidly fluctuate between these two regions, as shown in Figure S11. Additionally, the larger amount of time for which ions are found close to the interface compared to in the bulk (see Table S4), suggests a better statistical significance of those results.

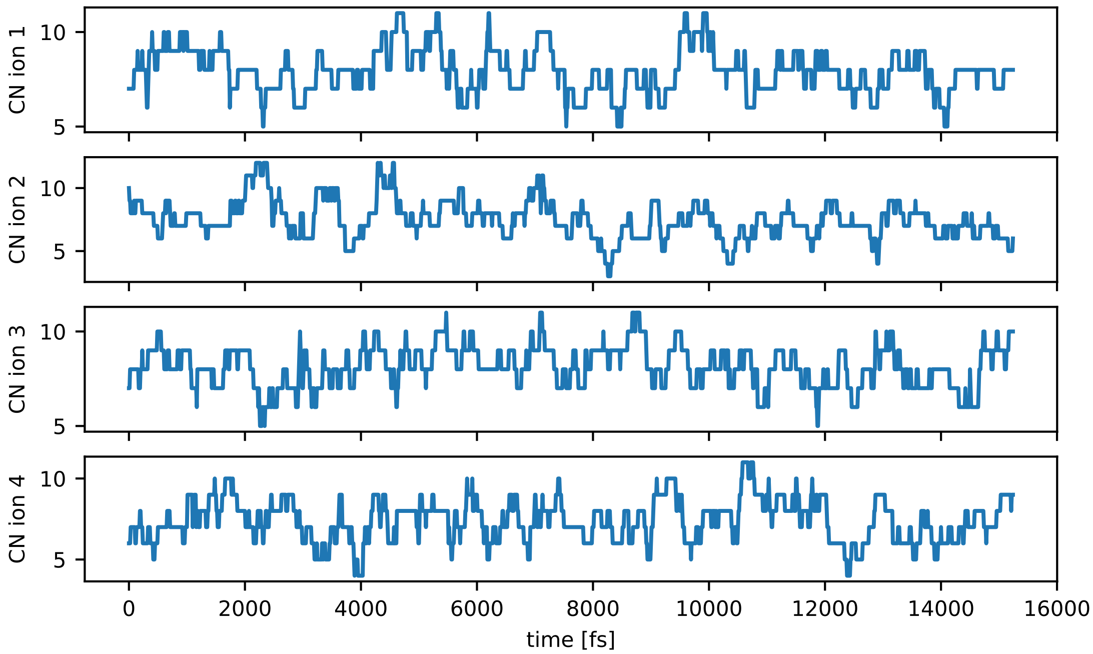

Figure S10: Ion-water coordination number (CN) over time for ions 1-4 in the simulation containing 4 ions. The cutoff radius for water molecules contributing to the ion-water coordination number is set to 3.999 Å.

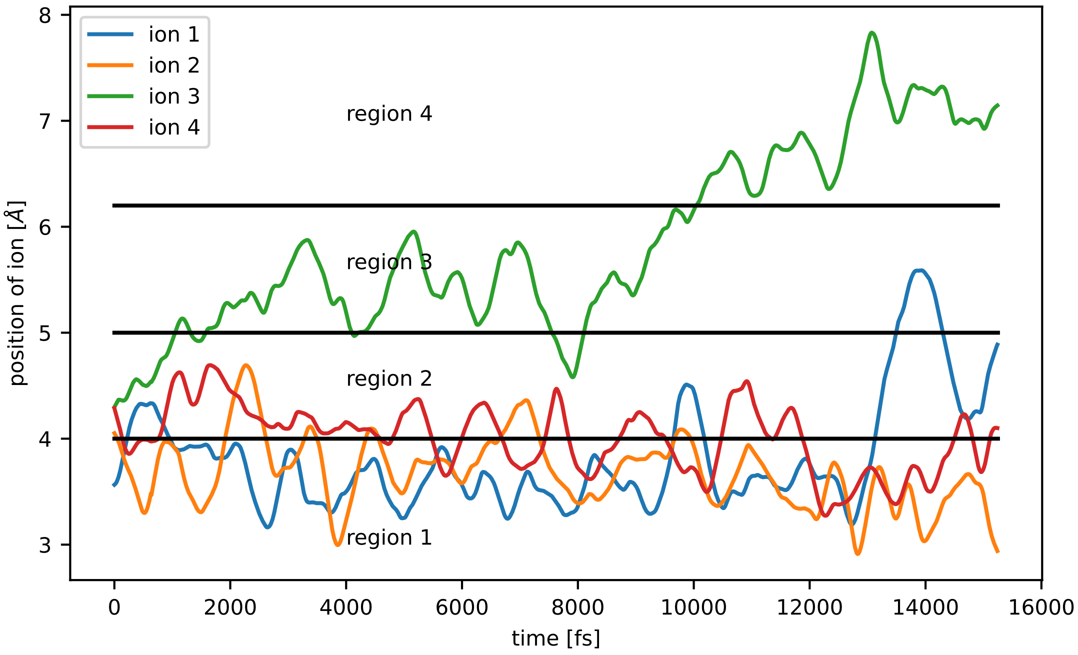

Figure S11: Ion position over time for the 4 ions in the simulation containing 4 ions. Ions 2 and 4 oscillate between region 1 and 2; Ion 1 mainly remains in region 1 and ion 3 resides mainly in regions 3 and 4.

Table S4: Average coordination number obtained for different simulations and ions in different regions. The cutoff radius is set to 3.999 Å. Additionally, the overall time spent by ions in a particular region is listed, to be able to assess the statistical relevance of a number cited.

	**Coordination number**			**Overall time spent in region [ps]**		
**Range/Simulation**	**4 Cs**	**2 Cs**	**Cs in bulk**	**4 Cs**	**2 Cs**	**Cs in bulk**
**0 Å-4 Å**	7.4	7.2	-	32	1	-
**4 Å-5 Å**	8.2	8.2	-	15	3	-
**5 Å-6.2 Å**	8.2	9.5	-	9	12	-
**6.2 Å-**	7.8	8.9	8.8	5	17	10

1.5 Opening and distortion of the solvation shell, and desolvation

1.5.1 Maximum opening angle for an undistorted solvation shell

We compute the maximum opening angle $2\theta_o^{max}$ for an ion with an undistorted, but optimally oriented solvation shell. This is shown schematically in Figure S12 for a particle with 6 water molecules in the solvation shell. In this case, the optimal packing is an octahedral, and $\theta_o^{max} \approx 55°$.

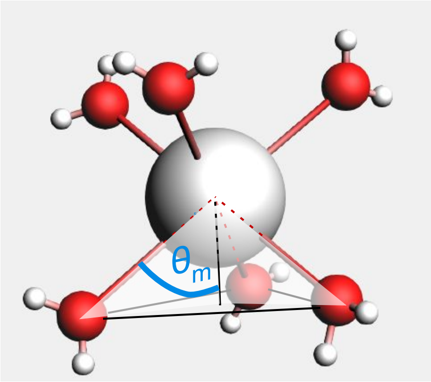

Figure S12: Schematic of the maximum opening angle after optimal orientation of an octahedral solvation shell.

For ions with an arbitrary number of water molecules, the optimal packing cannot be uniquely defined. Here, we use the spiral points method as proposed in Saff and Kuijlaars[4] to distribute our water molecules. For $n = 6$, as used above, we then find $\theta_o^{max} = 61°$. For $n = 8$, we find $\theta_o^{max} \approx 54°$. A coordination number of $n = 8$ corresponds closely to the coordination number found in our simulations for a cutoff distance $r_{cut} = 3.999$ Å, which is the cutoff distance used to obtain the results in Figure 4d in the main text. This allows us to compare our predictions for the maximum opening angle (after optimal orientation of the solvation shell) to the average angle distribution shown in Figure 4d and the distribution of the minimum opening angle θ_{min} for ions at different distances from the surface as shown in Figure S13.

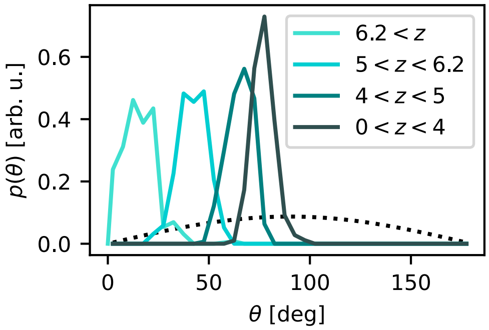

Figure S13: Similar to Figure 4e in the main text, but only showing the minimum of all angles θ per ion. This corresponds to half the opening angle θ_o.

For ions residing at 5 Å $< z < 6.2$ Å, we find the half opening angle between $\theta_o = 25°$ and $\theta_o = 60°$, smaller than the maximum opening angle computed above. This suggests that the solvation shell in for ions at this distance from the surface does not have to be optimally oriented yet; a slight orientation is clearly indicated by θ being considerably larger than zero though.

For ions residing between 4 Å $< z < 5$ Å, the half opening angle increases to $\theta_o = 45°$ and $\theta_o = 85°$, with a peak at $\theta_o \approx 67°$. This angle is larger than the maximum half opening angle computed above for $n = 8$ above, suggesting that the solvation shell of ions residing in this region is not only strongly

oriented but that, additionally, the number of water molecules in the solvation shell must either be decreased or the solvation shell must be distorted. Within our simulations, we do not find the coordination number to decrease for ions at $4\,\text{Å} < z < 5\,\text{Å}$ (see discussion below), but we find the solvation shell to be distorted, as shown in Figure S14, which shows the O-ion-O angles formed by the simplices of the hull formed by O atoms in the solvation shell. These angles can be used as a measure of O-O nearest-neighbor distances. For ions residing between $4\,\text{Å} < z < 5\,\text{Å}$, we see a clear shift towards lower angles, i.e., towards a closer packing of the oxygen atoms in the region where oxygen atoms reside.

For ions residing even closer to the surface at $z < 4\,\text{Å}$, the opening angle increases even further, with peak values of $\theta_o \approx 77°$. We do not see a further shift towards smaller O-ion-O angles in Supplementary Figure 14, but we observe a loss in probability to find large O-ion-O angles along the simplices of the O hull of the ions. This indicates that the ion desolvates. Our results (presented below) indeed suggest a slight desolvation. This slight desolvation is sufficient to explain the large opening angles found in this case: Eliminating only one water molecule from the geometries obtained from the spiral points method above, while keeping the location of all other water molecules fixed, increases the maximum half opening angle to 75°.

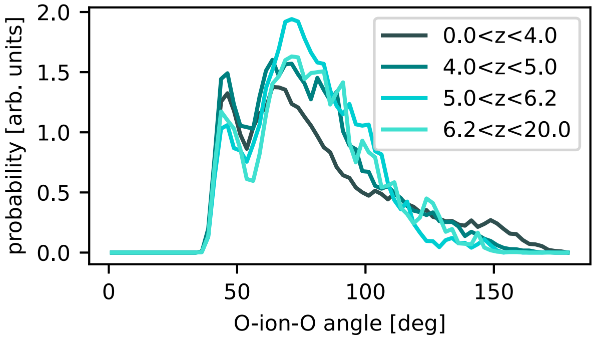

Figure S14: Probability distribution of O-ion-O angles of simplices formed by the hull of O atoms in the solvation shell of Cs for ions at different distances from the surface.

In summary, as the ion approaches the surface, the solvation shell first orients optimally towards the surface, then starts to distort and finally, the solvation is slightly shed.

1.5.2 Energetic considerations

To rationalize the partial desolvation of ions close to the interface, we estimate the gain in electrostatic energy obtained from moving the Cs ions closer to the surface as well as the expected energy penalty from (partially) desolvating the ions. To compute the gain in electrostatic energy gain achieved when moving all ions simultaneously closer to the interface, we model the interface as perfect capacitor.

We approximate the capacitance as:

$$C = \frac{\varepsilon_0 \varepsilon_r A}{d - d_0}$$

where, ε_0 is the vacuum permittivity, ε_r the dielectric constant, and $d - d_0$ is the distance of the ion from the image charge plane. We set distance of the image charge plane from the metal cores in the uppermost layer to $d_0 = 1$ Å and the dielectric constant $\varepsilon_r = 6$. Furthermore, we set d to the values found experimentally, namely $d_{high} = 4.6$ Å at high potentials and $d_{low} = 3.7$ Å at low potentials. This leads to plausible values for the capacitance between 15 $\mu F/cm^2$ and 20 $\mu F/cm^2$. We note that a larger ε_r would lead to a larger capacitance and a smaller electrostatic energy (gain).

To compute the electrostatic energy,

$$E_{elec} = \frac{1}{2}\frac{Q^2}{C}$$

we also require the free surface charge Q. In our simulations, we found a surface charge of $4e^-$ per surface area $A = 250$ Å^2 to lead to a potential of ~ –1 V vs. Ag/AgCl, in good agreement with the lowest experimentally applied potential. We use this surface charge to compute the change in electrostatic energy when moving the shielding ions from d_{high} to d_{low}, giving:

$$\Delta E_{elec} \approx 1000 \text{ meV}$$

Considering that there are four ions in the cell, this leads to a gain in electrostatic energy per ion when moving all shielding ions closer to the surface of:

$$\Delta E_{elec}^{ion} \approx 250 \text{ meV}.$$

Similarly, we could have considered moving only one ion closer to the interface, while keeping the others fixed at d_{high}. We use the same assumptions as above to compute the potential vs. potential of zero charge for a simulation cell with 4 ions and $\frac{C}{A} = \frac{C_{high}}{A} = 15\frac{\mu F}{cm^2}$ via $\Delta U = \frac{Q}{C}$, leading to $\Delta U \approx 1.7$ V. (Note that this ΔU is likely somewhat too high when comparing to experimental values of the pzc, but results should not change by more than a factor 2). Moving an ion from d_{high} to d_{low} and taking the position d_{im} of the image charge plain into consideration, this should lead to an energy gain of:

$$\Delta \tilde{E}_{elec}^{ion} = \mathcal{E}\Delta d = \frac{\Delta U}{d_{high} - d_{im}}\left(d_{high} - d_{low}\right) \approx 400 \text{ meV}$$

where, $\mathcal{E}$ is the surface electric field. (Note again that this value is likely somewhat too high as $\Delta U = U$ (4 ions) $- U_{pzc}$ and hence $U_{pzc} = \Delta U - U(4 \text{ ions}) = 1.7 - 1 \text{ V vs}\frac{\text{Ag}}{\text{AgCl}} = 0.7 \text{ V vs.}\frac{\text{Ag}}{\text{AgCl}}$ overestimates U_{pzc} compared to experiment).

The electrostatic energy gain should be compared to the solvation enthalpy of Cs, which is given by $E_{solv} = 2.7$ eV. The results presented above suggest that a ~ 10% desolvation should be achievable when

moving the ion from $d_{high} = 4.6$ Å to $d_{low} = 3.7$ Å, consistent with the statements made in the main text. For smaller surface charges, the electrostatic energy gain will be smaller, possibly hindering partial desolvation, explaining why ions reside further from the surface at potentials closer to the point of zero charge (PZC).

1.6 Diffusion of Cs ions

In the following, we show that the Cs ion position is not thermodynamically equilibrated within the timescale of our simulations. So show this, we present results here of five different calculations in which a single Cs atom was initialized at various different distances from an Au(111) surface. The initial positions are highlighted by the vertical dashed lines in Figure S15. In all cases in which the ion was released far from the surface, the ion passed a significant distance within the first 5 ps of the simulation, which we consider as equilibration time and which are not included in the histograms in Figure S15. The ion can thus diffuse rather readily in the simulation cell. Nevertheless, two of the 5 simulations show a significantly different ion position probability with the ions situated far further from the surface than in the other 3 simulations. This clearly shows that the ions do not fully thermalize within the AIMD timescales. The average ion position and the probability distribution of where to find an ion should therefore not be used as output of the AIMD simulations.

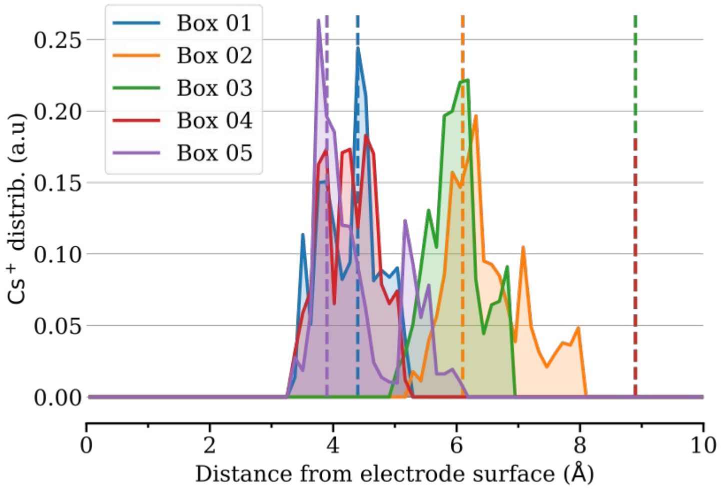

Figure S15: 5 different simulations for a Cs ion above an Au(111) surface. Each simulation has different initial geometries for the water and the initial position of the ion is varied. Dashed lines indicate the starting position of the ion, the histograms show the probability of where to find the ion after 5 ps of equilibration in a 15 ps simulation run (i.e, 10 ps of statistics accumulation).

2 Supporting Methods

2.1 Experiments

An upside-down hanging meniscus electrochemical cell was used for the SXRD measurements as described elsewhere.[5] A syringe pump was used to deliver electrolyte to the cell, control the volume and the meniscus size. The meniscus was constantly monitored using a camera, such that a as constant as possible meniscus size was maintained. This could also be double checked by looking at the electrochemical current (see Figure S6a in the SI). The electrochemical signal was controlled and recorded using a Bio-Logic potentiostat/galvanostat/EIS (SP-300). Prior to the experiments, all parts of the setup that meet the electrolyte were stored in potassium permanganate solution (1 g L^{-1} $KMnO_4$ dissolved in 0.5 M H_2SO_4) overnight and cleaned by immersion in dilute piranha (3:1 v/v mix of H_2SO_4 (96%)/H_2O_2 (30%), diluted with water). The parts were then boiled at least five times in ultrapure water (>18.2 MΩ·cm). Part of the syringe pump components (syringes and tubing) were not boiled but flushed extensively with hot ultrapure water.

The Au(100) sample (Surface Preparation Laboratory - SPL, cut and polished <0.1°) was prepared by several cycles of mild etching (±2 V vs. Au wire for 10 s followed by immersion in a 5 M HCl solution), flame annealing with a butane torch, and cooling in an argon atmosphere. In between measurements, the electrode was flame annealed and the surface kept in a controlled atmosphere, before the electrode was mounted in the SXRD cell. The alignment of the sample was always performed without electrolyte on the surface and while flushing the cell enclosure with Ar. The electrolytes were prepared using the following chemicals: $HClO_4$ (99.999% trace metals basis) and $CsClO_4$ (Sigma-Aldrich, 99.99%, trace metal basis) for experiments at pH 3, and CsOH (Sigma-Aldrich, 99.95%, trace metal basis) for pH 13. The Cs^+-containing electrolytes were purified by long-term electrolysis prior to the experiments. Two large surface area gold electrodes were used, and a potential of −2 V was applied for 12 h, similarly to the procedure reported elsewhere.[6] Prior to and during the experiment, the electrolyte is severely purged with Ar. A leakless miniature Ag/AgCl (ET072-1) was used as reference and a gold wire as counter electrode. Note that for every other CTR data shown in Figure 2, a freshly prepared, thermally reconstructed sample was used, and that potentials where the reconstruction was lifted were avoided.

2.2 Simulations

The Au (100)-*hex* surface was built by fitting a (111)-hexagonal surface layer onto a (100)-orientated electrode and by approximating the buckling of the hexagonal surface layer following the work of Jacob.[7]A four-layer slab (in the z-direction) was built for which the top (resp. bottom) two layers were relaxed (resp. kept in their bulk position) to simultaneously mimic the surface relaxation and bulk behavior of the electrode. A lattice constant of 4.08 Å was used to build the surfaces, as obtained from our Au bulk optimization calculation, and consistent with the experimental value.[8] The slabs were separated by 40 Å, the lower ~ 20 Å were filled by 154 water molecules, leaving ~ 20 Å of vacuum. The water region was chosen long enough to minimize the error on the calculation of the electrode potential[9] and to leave

sufficient space for the ions to move around. The vacuum layer should avoid excessive interactions between periodic images of the surfaces and allows for the use of a surface dipole correction which was applied in the z-direction of the cell to mitigate spurious interactions between periodic images caused by the intrinsic dipole moment of the asymmetric slab geometry. Due to the large size of the simulation cells, *i.e.*, 14.4 x 17.3 x 48.6, only the Γ point in the reciprocal space was used for all the simulations.

The simulation cell was pre-equilibrated for 10 ns for the halved cell at the force-field level by using the code MetalWalls.[10] The SPC/E force field was used for water,[11] while the Au atoms were described by a force field proposed by Heinz et al.[12] After this force-field pre-equilibration period, ions were inserted into the (halved) unit cells and an AIMD pre-equilibration was performed for 15 ns. The simulation cells were then doubled (to give the above dimensions), and further equilibrated for 5ps. Data was collected for another 15ps. Varying the amount of Cs^+ ions within the electrolyte region allowed the modification of the surface charge density, and, consequently, the electrode potential, which was extracted based on the computational standard hydrogen electrode (cSHE) approach[9] using the water bands in the bulk water region. Simulation cells with 2 and 4 Cs^+ ions lead to an electrode potential of –0.47 and –0.83 V vs. SHE, *i.e.*, –0.67 and –1.03 V vs. Ag/AgCl, respectively. AIMD simulations were conducted with thCP2K/Quickstep package,[13] in the NVT ensemble with a 1 fs time step employing a CSVR (canonical sampling through velocity rescaling) thermostat with a target temperature set at 360K. This temperature has been shown to compensate for the water over structuration of PBE-D3 functional and to accurately recover the experimental water bulk diffusion coefficient of 0.22 Å^2/ps.[14] A reference Cs^+ bulk simulation was conducted by inserting one Cs^+ ion within a 15 x 15 x 15 cubic cell filled with water.

2.2.1 Hydrogen bonding network

Hydrogen bonds are defined according to Wang et al.[15], i.e., if the oxygen donor-oxygen acceptor distance $d_{O_d \to O_a} < 3.5$ Å, and the oxygen acceptor-oxygen donor-hydrogen donor angle $\alpha_{O_a \to O_d \to H_d} < 30°$.

2.2.2 O-ion-O angles along simplices

To quantify the possible distortion of the solvation shell, we analyze the oxygen-ion-oxygen angles for oxygen atoms in the first solvation shell along simplices of the hull formed by the oxygen atoms in the first solvation shell. To this end, we first define all oxygen atoms in the first solvation shell and project them onto a sphere with unit radius. This step is necessary to allow for the use of a convex hull algorithm without "loosing" oxygen atoms that are situated somewhat closer to the ion. We then use the convex hull algorithm present in the python package scipy.spatial to define the simplices connecting oxygen atoms building the convex hull. For all oxygen atoms connected by an edge of the simplices, we compute the oxygen-ion-oxygen angle.[12,13]

References

(1) Ocko, B. M.; Wang, J.; Davenport, A.; Isaacs, H. In Situ X-Ray Reflectivity and Diffraction Studies of the Au(001) Reconstruction in an Electrochemical Cell. *Phys Rev Lett* **1990**, *65* (12), 1466–1469.

(2) Havu, P.; Blum, V.; Havu, V.; Rinke, P.; Scheffler, M. Large-Scale Surface Reconstruction Energetics of Pt(100) and Au(100) by All-Electron Density Functional Theory. *Phys Rev B Condens Matter Mater Phys* **2010**, *82* (16), 1–4.

(3) Peng, L.-M.; Ren, G.; Dudarev, S. L.; Whelan, M. J. Debye–Waller Factors and Absorptive Scattering Factors of Elemental Crystals. *Acta Crystallogr A* **1996**, *52* (3), 456–470.

(4) Saff, E. B.; Kuijlaars, A. B. Distributing Many Points on a Sphere. *The mathematical intelligencer* **1997**, *19* (1), 5–11.

(5) Magnussen, O. M.; Krug, K.; Ayyad, A. H.; Stettner, J. In Situ Diffraction Studies of Electrode Surface Structure during Gold Electrodeposition. *Electrochim Acta* **2008**, *53* (9), 3449–3458.

(6) Li, X.; Gunathunge, C. M.; Agrawal, N.; Montalvo-Castro, H.; Jin, J.; Janik, M. J.; Waegele, M. M. Impact of Alkali Metal Cations and Iron Impurities on the Evolution of Hydrogen on Cu Electrodes in Alkaline Electrolytes. *J Electrochem Soc* **2020**, *167* (10), 106505.

(7) Jacob, T. Potential-Induced Lifting of the Au(1 0 0)-Surface Reconstruction Studied with DFT. *Electrochim Acta* **2007**, *52* (6), 2229–2235.

(8) Introduction to Solid State Physics Charles Kittel. **2005**.

(9) Le, J.; Iannuzzi, M.; Cuesta, A.; Cheng, J. Determining Potentials of Zero Charge of Metal Electrodes versus the Standard Hydrogen Electrode from Density-Functional-Theory-Based Molecular Dynamics. *Phys Rev Lett* **2017**, *119* (1), 016801.

(10) Marin-Laflèche, A.; Haefele, M.; Scalfi, L.; Coretti, A.; Dufils, T.; Jeanmairet, G.; Reed, S. K.; Serva, A.; Berthin, R.; Bacon, C.; Bonella, S.; Rotenberg, B.; Madden, P. A.; Salanne, M. MetalWalls: A Classical Molecular Dynamics Software Dedicated to the Simulation of Electrochemical Systems. *J Open Source Softw* **2020**, *5* (53), 2373.

(11) Berendsen, H. J. C.; Grigera, J. R.; Straatsma, T. P. The Missing Term in Effective Pair Potentials. *Journal of Physical Chemistry* **1987**, *91* (24), 6269–6271.

(12) Heinz, H.; Vaia, R. A.; Farmer, B. L.; Naik, R. R. Accurate Simulation of Surfaces and Interfaces of Face-Centered Cubic Metals Using 12-6 and 9-6 Lennard-Jones Potentials. *Journal of Physical Chemistry C* **2008**, *112* (44), 17281–17290.

(13) Vandevondele, J.; Krack, M.; Mohamed, F.; Parrinello, M.; Chassaing, T.; Hutter, J. Quickstep: Fast and Accurate Density Functional Calculations Using a Mixed Gaussian and Plane Waves Approach. *Comput Phys Commun* **2005**, *167* (2), 103–128.

(14) Liu, L. M.; Krack, M.; Michaelides, A. Interfacial Water: A First Principles Molecular Dynamics Study of a Nanoscale Water Film on Salt. *Journal of Chemical Physics* **2009**, *130* (23).

(15) Wang, R.; Dellostritto, M.; Klein, M. L.; Borguet, E.; Carnevale, V. Topological Properties of Interfacial Hydrogen Bond Networks. *Phys Rev B* **2024**, *110* (1), 014105.
